# StORF-Reporter: finding genes between genes

**DOI:** 10.1093/nar/gkad814

**Published:** 2023-10-28

**Authors:** Nicholas J Dimonaco, Amanda Clare, Kim Kenobi, Wayne Aubrey, Christopher J Creevey

**Affiliations:** Institute of Biological, Environmental and Rural Sciences, Aberystwyth University, Aberystwyth SY23 3PD, Wales, UK; Department of Computer Science, Aberystwyth University, Aberystwyth SY23 3DB, Wales, UK; Department of Medicine, McMaster University, Hamilton, ON, Canada; Farncombe Family Digestive Health Research Institute, McMaster University, Hamilton, ON, Canada; School of Biological Sciences, Queen’s University Belfast, Belfast BT7 1NN, Northern Ireland, UK; Department of Computer Science, Aberystwyth University, Aberystwyth SY23 3DB, Wales, UK; Department of Mathematics, Aberystwyth University, Aberystwyth SY23 3BZ, Wales, UK; Department of Computer Science, Aberystwyth University, Aberystwyth SY23 3DB, Wales, UK; School of Biological Sciences, Queen’s University Belfast, Belfast BT7 1NN, Northern Ireland, UK

## Abstract

Large regions of prokaryotic genomes are currently without any annotation, in part due to well-established limitations of annotation tools. For example, it is routine for genes using alternative start codons to be misreported or completely omitted. Therefore, we present StORF-Reporter, a tool that takes an annotated genome and returns regions that may contain missing CDS genes from unannotated regions. StORF-Reporter consists of two parts. The first begins with the extraction of unannotated regions from an annotated genome. Next, Stop-ORFs (StORFs) are identified in these unannotated regions. StORFs are open reading frames that are delimited by stop codons and thus can capture those genes most often missing in genome annotations. We show this methodology recovers genes missing from canonical genome annotations. We inspect the results of the genomes of model organisms, the pangenome of *Escherichia coli*, and a set of 5109 prokaryotic genomes of 247 genera from the Ensembl Bacteria database. StORF-Reporter extended the core, soft-core and accessory gene collections, identified novel gene families and extended families into additional genera. The high levels of sequence conservation observed between genera suggest that many of these StORFs are likely to be functional genes that should now be considered for inclusion in canonical annotations.

## Graphical Abstract

**Graphical Abstract:**
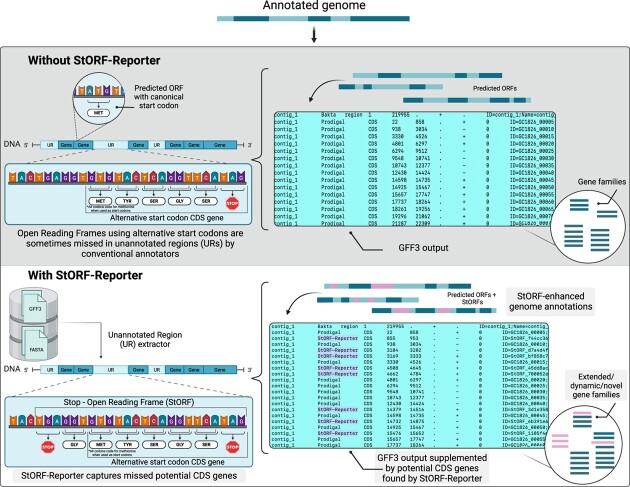

## Introduction

Prokaryotic genomes are most often observed to have high levels of gene density, resulting in low proportions of unannotated DNA, especially compared to eukaryotes (1,2). Much of this unlabelled DNA is intergenic, i.e. found between annotated genomic elements such as genes, promoters or other functionally and structurally important regions. In eukaryotes, the relative proportion of a genome reported to be intergenic has been linked to the biological complexity of the organism (3). However, in prokaryotes, perceived intergenic regions are often assumed to be not functionally important, despite studies reporting that intergenic regions contain putative coding and non-coding genes (complete and pseudogenised) (4–6).

Recently, evidence has emerged of elevated levels of conservation and selection in putative intergenic regions (7) suggesting that these regions should be investigated to determine whether they contain genes or other functional elements missed by contemporary prediction methods. Evidence of missed genes has also been demonstrated via the results of ribosome profiling using Ribo-seq and proteomic detection using mass spectrometry. These techniques have recently discovered, for example, short ORFs that were missed by *in silico* annotation tools (8,9). However, these lab-based methods have their limitations, requiring the right growth conditions to capture the expression or production of proteins. Ribo-seq requires suitable lysis of the cells, and pure cultures in very large volumes, and while Ribo-seq for microbiomes is in development (10) this is not as successful as for cultured cells. Proteomics requires extensive databases with which to compare sequences. Ribosome profiling and proteomics are also much more expensive and time-consuming than computational methods. There are tools such as iPtgxDBs (11) that promise to integrate multiple reference annotations with *ab initio* predictions and *in silico* ORFs, with the aim to ‘cover[s] the entire protein-coding potential of a prokaryotic genome’. However, as with all tools that rely on proteogenomics and other experimental evidence, it is not always straightforward to evaluate the results (12) or ascertain a truly complete picture of the protein coding potential of any organism. Therefore, in this work, we investigate *in-silico*, the potential for prokaryotic ‘intergenic regions’, (referred to from this point on as *unannotated regions* (URs) for clarity) to harbour CDS genes that are most-often missing from canonical annotations and also those yet-to-be-discovered.

**Figure 1.**
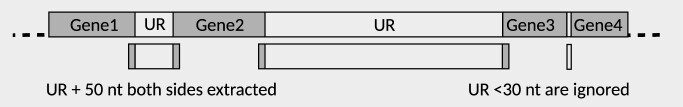
Visual representation of how unannotated regions (URs) are selected for extraction. URs that are less than 30 nt are not extracted. URs are extracted with an additional 50 nt on their 5’ and 3’ ends to allow for overlapping genes and the upstream untranslated region between the first stop codon and the true start codon.

Computational methods employed to study URs have often relied on sequence similarity to known proteins deposited in databases and, as such, are limited to the set of proteins already discovered and by the thresholds used to identify a match. Therefore, to investigate these regions and overcome these and other limitations inherent to the identification of coding regions in prokaryotic genomes (2), we have developed a novel methodology to identify and extract putative CDSs in the URs of existing prokaryotic genome annotations. This approach, named ‘StORF-Reporter’, is carried out in two stages. First, URs are extracted from an annotated genome (see Figure 1), then we identify and filter from these extracted URs open reading frames (ORFs) that are delimited by two in-frame stop codons (termed StORFs for Stop-ORFs).

Conflicting definitions of an ORF exist in the literature (13), and while it is the norm for prokaryotic annotation tools to annotate an ORF as the region between a start and stop codon in a coding sequence, it should be noted that the Sequence Ontology (14) does not make reference to a start codon in its definition of an ORF. Instead, it is described as “the in-frame interval between the stop codons of a reading frame which when read as sequential triplets, has the potential of encoding a sequential string of amino acids”. Therefore, how we define a StORF is synonymous with the definition of an ORF in the Sequence Ontology, but for clarity and due to the prevalence of the start-to-stop definition of an ORF in the literature, we refer to stop-to-stop open reading frames as StORFs hereafter.

**Figure 2.**
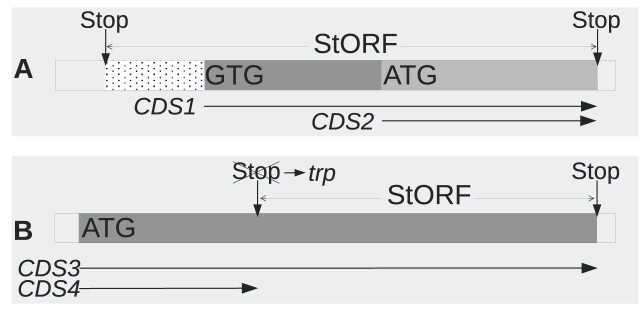
Visual representation of a StORF and how it can capture multiple potential start codons in an unannotated region. Part **A** depicts a StORF capturing two possible start positions (GTG and ATG) for a CDS gene which could produce two distinct CDS sequences (*CDS1* and *CDS2*). The dotted segment of the StORF represents the untranslated part of the sequence. Part **B** shows how a StORF can comprise of only a partial segment of a gene if that gene either recodes a canonical stop codon (Stop to trp) or has had an in-frame stop codon mutation resulting in one complete and one truncated transcript (*CDS3* and *CDS4*). StORF-Reporter can be used to find these consecutive StORFs.

An example of a StORF can be seen in Figure 2, which captures multiple possible start codons. The choice of which start codon to use as the start of the coding region in a situation like this will usually come down to the specific weightings inherent in the tools used and/or the matches they received to any databases that are used in the annotation process. This quite often results in non-standard start codons being under-represented in prokaryotic annotations (2). Shifting the focus from locating the most likely start codon for an ORF to identifying the first upstream in-frame stop codon simplifies the problem as it reduces the dependency on correctly identifying genomic features such as the Shine–Dalgarno motifs (15), promoter regions (16), ribosomal binding sites and operons (17). It also reduces the potential for inadvertently truncating the predicted protein product by incorrectly choosing between alternative putative start codons (see Figure 2 A). This is important as while the canonical start codon ‘ATG’ is most often observed to initiate approximately 80% of prokaryotic CDS genes, many species and cross-species gene families have been shown to possess very different start codon profiles (18,19). Furthermore, there is growing evidence of the existence of organisms that adjust their genetic code in response to changing environments (20), thus making traditional start codon identification even more complex and giving rise to software that tries to consolidate differing annotations in order to resolve this (21). Unlike start codons, the three canonical stop codons (TGA, TAG, TAA) are the terminus of almost all CDS genes in almost all species, and despite the possible influence of purifying and/or positive selection (22,23), they can be reliably used to identify the terminus of protein synthesis in a gene. Additionally, it has been observed that stop codons appear more frequently than expected in non-coding reading frames, possibly due to selection against the translation of the wrong frame following frameshift mutations or ribosomal slippage (24). Thus, the longest StORF identified from all 6 reading frames in any UR has the greatest potential to represent a coding region.

We propose the StORF-Reporter methodology as an additional step to supplement contemporary prokaryotic genome annotations from *de novo* annotation workflows (such as Prokka (25) and Bakta (26)) or from reference databases (such as Ensembl (27)) in order to identify overlooked putative coding regions. As such, we provide tools to supplement existing genome annotation files with information on any StORFs identified. Therefore, the resulting enhanced annotation builds upon the work that proceeded it rather than restarting annotations from scratch.

To demonstrate the utility of this approach, we identified and investigated StORFs from three increasingly complex sets of genomes. First, we examined six bacterial model organisms that exhibit many URs long enough to contain full-length CDS genes, expanding the narrative of these well-studied genomes.

Second, we investigated the presence of conserved StORFs across 169 *E. coli* strains elucidating their impact on our understanding of the *E. coli* pangenome.

Lastly, we applied the methodology to 5109 prokaryotic genomes representing 247 genera from Ensembl Bacteria to understand the trends of StORFs across diverse bacterial groups.

## Materials and methods

### Datasets

Six bacterial model organisms; *Bacillus subtilis* BEST7003 strain, *Caulobacter crescentus* CB15 strain, *E. coli* K-12 ER3413 strain, *Mycoplasma genitalium* G37 strain, *Pseudomonas fluorescens* UK4 strain, *Staphylococcus aureus* 502A strain, shown in Table 1, were selected for the first study.

**Table 1.** An overview of genome composition for the six model organisms used in the analysis of StORF-Reporter

Model organism	Genome size (Mb)	GC content (%)	Genes **[density]**
***B. subtilis*** (BEST7003)	4.04	43.89	4133 **[88.91%]**
***C. crescentus*** (CB15)	4.02	67.21	3875 **[90.60%]**
***E. coli*** K-12 (ER3413)	4.56	50.80	4257 **[86.28%]**
***M. genitalium*** (G37)	0.58	31.69	559 **[92.03%]**
***P. fluorescens*** (UK4)	6.06	60.13	5266 **[84.75%]**
***S. aureus*** (502A)	2.76	32.92	2556 **[83.93%]**

This table shows the number of Ensembl-annotated genes (coding and non-coding). The gene density is presented in bold square brackets. These six organisms span a range of genome size (0.58–6.06 Mb) and gene density (percentage covered with annotation, 83.93–92.03%).

The canonical sequence and annotation data for 31 332 genomes (29 900 bacteria and 1213 archaea) were acquired from the 55th release of Ensembl Bacteria (27). For each genome, three data files were downloaded; the complete DNA sequence (*_dna.toplevel.fa), the annotated protein sequences (*.ṗep.all.fa) and the GFF (Generic Feature Format) file (*.gff3) containing the position information for each gene (coding and non-coding). The genomic elements presented in the Ensembl GFF annotations were taken as reference annotations for this study. Fragmented genomes were removed by filtering out those with more than 5 contigs (not including plasmids). Then, genera that had less than 5 genomes were also removed. This resulted in 5109 genomes (see Supplementary Table 1).

Additionally, the *E. coli* species was selected to undergo a pangenomic analysis of its Ensembl annotated genes and supplemented StORFs. As many of the Ensembl genomes were unlabelled at the species level, to make sure the correct genomes were extracted, only those labelled specifically as ‘*Escherichia_coli*’ were extracted. This resulted in a group of 173 *E. coli* genomes from the original filtered group of 5109. These were further filtered using sourmash (28) and GTDB-TK (29) to ensure these were true *E. coli* genomes. This resulted in a usable dataset of 169 *E. coli* genomes for pangenomic analysis (see Supplementary Table 2 for further detail). Concerningly, at least one of the 4 removed genomes (Escherichia_coli_gca_900636015.38490) had already been flagged by ENA as mislabelled in 2020 but this is yet to be reflected in the Ensembl database v55.

**Table 2.** Table containing the number of StORFs found in the unannotated regions recovered from Ensembl annotations for the six model organisms

Model organism	# URs/StORFs	Swiss-Prot [Cov ≥ 80%]	Intra-Genome [Cov ≥ 80%]
*B. subtilis*	2711/2582	28 **[23]**	6 **[1]**
*C. crescentus*	2321/1707	6 **[4]**	14 **[2]**
*E. coli*	2743/3036	160 **[119]**	58 **[13]**
*M. genitalium*	157/180	70 **[3]**	58 **[0]**
*P. fluorescens*	3509/3633	71 **[44]**	73 **[39]**
*S. aureus*	1666/2289	22 **[4]**	16 **[3]**

The numbers of StORFs with a high sequence similarity to a Swiss-Prot protein or the Ensembl proteome of their model organism are listed. In addition, those StORFs with a high similarity and also ≥80% subject coverage (Cov) are reported in brackets in bold.

### StORF-Reporter

To identify StORFs, we developed the ‘StORF-Reporter’ annotation-enhancement platform. StORF-Reporter contains a number of distinct sub-packages that can be used together or independently as they were in this study. The sub-packages UR-Extractor and StORF-Finder were used, in order, to extract URs and report StORFs for each of the genomes in this study. A third tool, StORF-Reporter is available to perform UR and StORF extraction from most canonical GFF annotation formats, or Prokka or Bakta annotated genomes, and presents the user with a newly created canonical or Prokka/Bakta formatted GFF file supplemented with StORFs for use in downstream tools that require the those specific formats. Additionally, StORF-Reporter can perform *de novo* CDS prediction using Pyrodigal (30,31), and then StORF reporting if presented with an unannotated genome. Lastly, the StORF-Extractor sub-package can be used to extract the StORFs from an enhanced annotation and output them as either a FASTA file containing their DNA/protein sequence or a standalone GFF annotation file.

The processes of UR-Extractor and StORF-Finder are described in the following sections, and the all code is available at https://github.com/NickJD/StORF-Reporter.

#### Unannotated Region-Extractor (UR-Extractor)

To facilitate the extraction of URs from different prokaryotic genomes (and thus the various interpretations of the GFF format), UR-Extractor was developed. Written in Python3 (32) and using user-definable genomic features in GFF format to identify the boundaries of genomic elements, regions of DNA without annotation were extracted from the provided DNA sequence files. The output consists of a GFF3 and FASTA file with the details of the sequence and loci for each UR that can be used further analysis (see Supplementary Section 3.1 for the command line menu of UR-Extractor).

**Figure 3.**
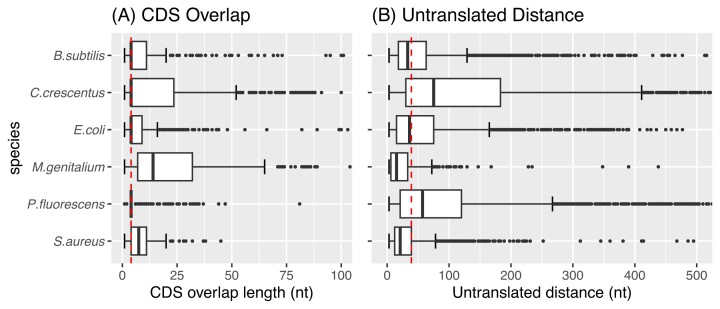
This double plot reports the analysis of the 6 model organisms which were used during the parameterisation of StORF-Reporter. Figure **A** reports the distributions of the Ensembl annotated CDS gene overlap lengths for each model organism with a dotted red line representing the overall median value of 4 nt, with the x-axis truncated at 100 nt. Figure **B** reports the distance in nucleotides between an Ensembl gene’s start codon and the first in-frame upstream stop codon for each model organism, with the x-axis truncated at 500 nt. The dotted red line represents the overall median value of 39 nt. This analysis indicates that an extension size of 50 nt from each end of the extracted unannotated regions is large enough to capture both the true overlap between an annotated gene and the putative gene identified by a StORF and the small amount of upstream non-coding DNA which the StORF will contain.

When recovering URs from a genome, it is important to consider that StORFs may overlap with annotated genes (33). Therefore, it was necessary to determine the extent to which the URs should be extended into the annotated regions. The 5109 genomes from Ensembl Bacteria, and the six model organisms in Table 1, were studied to identify representative parameters for the extraction of URs across this large set of annotated bacteria genomes. The median CDS gene overlap observed for each of the six genomes was 4 nt and most of the overlaps were below 50 nt, as can be seen in the left half (A) of Figure 3. The lengths of overlap between genes (both coding and non-coding) across the 5109 prokaryotic genomes were investigated and, as can be seen in Supplementary Figure S1, the gene overlap lengths observed for the 6 genomes are similar to the majority of the Ensembl annotated genomes. Furthermore, the median distance from a gene’s canonical start codon to the nearest in-frame upstream stop codon (representing the untranslated region of a StORF) was 39 nt (see the right half (B) of Figure 3). These findings demonstrate that StORFs should contain very little extraneous upstream DNA.

To account for the observed median gene and StORF features, URs were only extracted if they were at least 30 nt long but were extended by 50 nt at both the 5-prime and 3-prime ends (into annotated genes) by the UR-Extractor tool (see Figure 1), totalling a minimum length of 130 nt. This extension of 50 nt at both ends was to allow for the capture of the parts of StORFs which may overlap with existing gene annotations. This process also took into account the untranslated region between the start of the StORF and the putative start codon of the protein sequence it captured and was necessary as the direction of a potential StORF is not known in advance (see Figure 2 for an illustration of how a StORF captures upstream DNA).

Genomic repositories, including Ensembl Bacteria, are known to contain varying levels of assembly and annotation completeness and error due to a number of constraints (34–36). It can also be the case that genomes have missing annotations as a result of human or software error. Therefore, UR-Extractor has a maximum UR length threshold of 100 kb. A genome with a 100 kb UR either warrants further investigation for biological interest or is likely to contain annotation error.

The parameters used for UR-Extractor are available in Supplementary subsection 3.1.

#### Stop-Open Reading Frame-Finder (StORF-Finder)

To identify StORFs, StORF-Finder starts by scanning the previously extracted URs (or alternatively any DNA sequence) for loci encapsulated by in-frame, user-definable stop codons (see Figure 2). All three stop codons are frequently present in intergenic regions and also within genes but out-of-frame, hypothesised to prevent incorrect frame reading of gene sequences (37). However, as with start codons, stop codons are differentially preferred by groups of genes and gene families both from within the same and across different species (38). Therefore, to investigate whether StORF-Finder should preferentially select one stop codon over another, a comparison was performed on the abundance of the triplets with their usage as stop codons across the Ensembl genomes. We can see in Supplementary Figure S2 that the codon TAA is often used more frequently than its distribution across the genomes suggests. Similarly, TGA is often used less frequently than its distribution across the genome would suggest in all 6 model organisms (with TGA never used as a stop codon in *M. genitalium* (39)). For TAG, the usage is mostly in line with the overall distribution across the genomes. Furthermore, there is clear evidence of stop codon preference and even repurposing for amino acids in species such as *M. genitalium* and specific gene-types which utilise their T/UGA and T/UAG codons (40) for selenocysteine (41) and pyrrolysine (42). Nevertheless, these cases are rare and it can be assumed that for the majority of prokaryotic genomes, the three canonical stop codons can be treated equally. The results of this analysis (see Supplementary Figure S2) show that while there are differences between the three stop codons, they are not universal and it is likely not beneficial to make generalisations.

While removing the need to identify the location of start codons, StORFs do have their own drawbacks. To be consistent with the universal genetic code, codon table 11, only the codon ATG is translated to methionine (M) in the StORF reporter output. This presents a problem when reporting StORFs since alternative start codons (i.e. codons other than ATG which initiate translation) are incorrectly assigned the amino acid represented in the codon table 11 and not methionine as is observed in the protein (43). This is a difficult problem to overcome since we cannot predict alternative translation start codons in advance. To mitigate this, StORF-Finder can report both the nucleotide and amino acid sequences in its output.

As with traditional CDS annotation methods, without any filtering, StORF-Finder can report a very high number of overlapping and nested StORFs, even for short regions of DNA. Therefore, a filtering step was developed to reduce the number of StORFs reported. This is done by first ordering all StORFs by their length in descending order and then iteratively removing the nested or shorter StORFs in cases where two StORFs overlap beyond the defined length of 50 nt. This is done individually for each set of StORFs for each UR and is intended to maximise the likelihood that recovered StORFs contain functional sequences. The remaining StORFs are reported in FASTA (DNA and/or amino acid form) and GFF format with both locus coordinates for their original position within the original genome sequence, and their position relative to the UR in which they were reported.

The parameters used for StORF-Finder are available in Supplementary subsection 3.2.

### Extracting StORFs from Prodigal and Ensembl annotations of the six model organisms

To investigate the ability of StORF-Reporter to add to and enhance contemporary annotations, URs and StORFs were extracted from the 6 model organisms. This was done using the existing annotations provided by Ensembl Bacteria and for a *de novo* annotation provided by running the Prodigal (30) genome annotation tool. The default parameters of UR-Extractor (-gene_ident “ID=gene”) were used to identify the complete set of genomic elements (coding and non-coding) which were then used to extract URs from the Ensembl-provided annotations. Prodigal was applied to the complete DNA sequences of the six model organisms and a novel CDS prediction was performed using the tool’s default parameters. As Prodigal only predicted CDSs, UR-Extractor used the CDS coordinates to extract URs (-gene_ident “CDS”).

### *Escherichia coli* pangenome analysis

To investigate the impact that StORFs may have on the pangenome of a well-studied prokaryotic model organism, the Ensembl protein sequences and StORFs extracted from a collection of 169 *E. coli* strains were studied.

Although gene loci information is often used to build a pangenome, many of the URs which potentially harbour the novel CDS genes that StORF-Reporter identifies are unlikely to be in the same location across all genomes. Additionally, the fragmented state of the majority of genomes held in Ensembl Bacteria (as is the case for many repositories) further adds to the necessity to use alternative techniques to identify the pangenome. Amino acid sequence similarity is an established metric used to group gene families. It is used by the clustering tool CD-HIT (version 4.8.1) (44), which does not require loci information. Amino acid sequences present in the *E. coli* genome were downloaded from Ensembl Bacteria (*.pep.all.fa) and combined into one FASTA file and gene-clustering was then undertaken with CD-HIT. Gene clustering was performed with the following parameters: aligned sequence identity threshold of 0.9 (90%), length difference cutoff of 0.6 (60%), and the ‘-g’ option was set to the ‘more accurate’ option (see Supplementary Subsection 3.4 for more details). The consistent use of the 0.6 length difference cutoff between clustered sequences allowed for the instances where the StORF sequences contained additional upstream non-coding DNA which would have hindered the clustering of the matching coding regions. The strict sequence identity threshold of 0.9 ensured that the resulting clusters were very similar across the regions where they did align.

The output of CD-HIT consists of two datafiles, a ‘.clstr’ (cluster) file containing the full sequence cluster metadata and a FASTA file containing the representative ID and sequence for each cluster. The CD-HIT cluster file, which consisted of the identified Ensembl gene clusters, was used as a baseline against which to compare the additional StORF sequences. The amino acid sequences of the *E. coli* StORFs recovered from the URs for the same set of 169 genomes were then combined with the previous CD-HIT output (FASTA) from the Ensembl proteins, which consisted of one representative sequence for each cluster (representatives are often the longer of the sequences in any one cluster). This combined FASTA file then underwent another round of CD-HIT clustering with the same parameters and produced the final *E. coli* pangenome datafiles (see Supplementary Figure S3 and Subsection 3.4 for more details on the files created and used in this process).

We are interested in gene clusters that are core to the pangenome (found in ≥99% of genomes), make up the soft-core (≥95% and ${<} 99\% $ of genomes) or are accessory (≥15% and ${<} 95\% $ of genomes). The results of these clusters are complex and have been classified differently with respect to the origins of each sequence set. As such they will be used throughout the rest of this paper and are referred to as follows:

- **Ensembl-Only -** refers to the clusters with only Ensembl annotated protein sequences produced in the first CD-HIT clustering step.
- **Ensembl-StORF -** refers to the clusters which contain at least one Ensembl annotated protein sequence and one StORF amino acid sequence.
- **StORF-Combined-Ensembl -** refers to the clusters where StORF amino acid sequences combined at least 2 or more Ensembl cluster representatives together.
- **StORF -** refers to the clusters in the Ensembl-StORF group that were categorised as core, soft-core, accessory or cross-genera gene families with only the StORF amino acid sequences being counted.
- **StORF-Only -** refers to the clusters which solely contain StORF amino acid sequences.

To interpret the output from CD-HIT, a number of Python3 scripts were developed and are available on the StORF-Reporter GitHub repository https://github.com/NickJD/StORF-Reporter. The tool CD-HIT_StORF-Reporter_Pangenome_Builder.py was built to first identify the Ensembl families for the 169 *E. coli* strains. Next, it then identifies whether any StORF sequences clustered with the representatives of these gene families, and also whether there were any StORF-Only gene families spanning multiple *E. coli* strains. This allows for the recording of unique strains/genomes that make up each gene family and therefore the ability to calculate their pangenomic status (core, soft-core, accessory). The StORF-Finder output sequence ID tag ‘StORF’ was used to distinguish between the Ensembl and StORF sequences.

It is difficult to predict what the result of clustering many thousands of protein sequences together could be. By enforcing fixed rules on the natural world, it is inevitable that certain cases will be unaccounted for. For example, multiple representative sequences from separate Ensembl gene family clusters could be combined into a new single cluster with the addition of StORF sequences. To account for this, the Ensembl clusters that were combined when StORF sequences were added were first recorded separately in the Ensembl gene cluster results, and then additionally recorded in their new combined clusters with the additional StORFs (see results rows 1 and 2 respectively in Table 3).

**Table 3.** *Escherichia coli* pangenome gene families calculated from the set of 169 genomes. Definitions of the gene family category are as follows: core-genes ≥99%, soft-core genes ≥95% to $< 99\%$ and accessory genes ≥15% and $< 95\%$

Cluster type	Core	Soft-Core	Accessory
Ensembl-Only	752	2078	3289
Ensembl-StORF	66	89	568
StORF-Combined-Ensembl	0	1	16
StORF	2	21	587
StORF-Only	127	359	3832

Gene families are only present in one category (except ‘StORF’) and Cluster types are described in the methods.

The parameters used for the CD-HIT clustering are available in Supplementary subsection 3.4.

### Extracting StORFs from 5109 Ensembl genomes

An important aspect of gene family research concerns their diversity and spread across different genera. The StORF-Reporter methodology was applied to the 247 genera collection of 5109 filtered Ensembl Bacteria genomes using the Ensembl annotations for the UR and StORF extraction. The diversity of assembly and annotation quality of these genomes presented a number of obstacles. As with the *E. coli* genome collection, even after filtering, the genomes in this study were often in a fragmented state with a number of low-quality regions containing large sections of ambiguous nucleotides. With this in mind, URs and StORFs were extracted individually for each of the 5109 genomes using default parameters. The resulting StORF-Finder outputs were then combined into a single FASTA file with their original genus and species name appended to the start of each sequence header. For example, the full genome name of ‘Enterobacter_cloacae_ecwsu1.asm23997v1’ was appended to the start of the protein name ‘AEW71445’ to make ‘Enterobacter_cloacae_ecwsu1.asm23997v1|AEW71445’. The same CD-HIT parameters and workflow were undertaken as for the *E. coli* pangenomic study. A modification of the previously developed single species script was built to handle multiple genera. This script records multiple levels of taxa information for each of the sequences such as genera, species and strain. However, as the analysis of the *E. coli* pangenome had already demonstrated StORF-Reporter’s ability to capture gene sequences at the species level, only the genera specific to each sequence are reported in this study.

### Validation of recovered StORFs

Different validation methods were required to determine what was being identified by StORF-Reporter. For the StORFs identified from within the URs of the Prodigal annotations, an extension to the ‘ORForise’ platform (2) was developed. ORForise was originally designed to compare annotations from different CDS prediction tools to a reference annotation. This extension, ‘StORForise’, was used to identify the Ensembl CDS genes missed by Prodigal that StORF-Reporter was able to recover. The default parameters of what classified a gene as ‘detected’ were taken from the ORForise package (a minimum of 75% coverage of an in-frame CDS prediction for a reference CDS gene). StORF-Reporter was only presented with the extracted URs according to the Prodigal CDS predictions. Therefore, mispredictions by Prodigal could result in the elongation of an Ensembl CDS gene or prediction of a CDS where no Ensembl annotation existed. These would constrain the available regions that could be searched in by StORF-Reporter.

Therefore, as part of the ORForise platform, two additional scripts were developed to identify the Ensembl genes that Prodigal missed and specifically the number of missed Ensembl genes which were non-vitiated (not corrupted or contaminated) by the mispredictions of Prodigal (false positives, overlapping Ensembl genes by more than 50 nt).

The protein sequence aligner DIAMOND (45) (version v0.9.30.131) was used to align the recovered StORFs from the Prodigal and Ensembl annotations of the six model organisms to known proteins from either the same genome’s proteome or from the Swiss-Prot protein database (46) (downloaded 12/10/2022). The sequence similarity alignments were performed with the DIAMOND blastp option with two separate parameter runs, one with a minimum bitscore of 60 and the second with the same bitscore but also a subject coverage cut-off of ≥80%. This was done to report whether some StORFs were the result of gene duplication or whether they had previously been identified in other studies. This was performed at the amino acid level as although the coding start sites are not identified by StORF-Finder, the coding frame is, and so the upstream region will include the start codon in the same frame (unless mutations are present). This indicates that StORFs can be directly translated into amino acid sequences, and then undergo similarity analysis to find homologous proteins already reported across other genomes and protein databases.

## Results

### Unannotated regions of model organisms

UR-Extractor was first applied to both the canonical Ensembl annotations and the Prodigal predictions of the six model organisms. Details of these URs can be seen in Supplementary Tables 3 and 4. These show that the numbers of URs reported for each model organism were similar between the Ensembl and Prodigal annotations, except for *M. genitalium* which were 157 and 636 respectively. This was likely due to the inability of Prodigal to account for the re-purposing of the ‘UGA/TGA’ stop codon causing a number of predicted CDSs to be incorrectly truncated. Additionally, the Ensembl annotations also included non-coding genes which were treated the same as coding genes by UR-Extractor. As Prodigal does not predict non-coding genes, it is likely to report additional or longer URs than those recovered using the Ensembl annotations. This can explain some of the longer UR lengths in the Prodigal analysis for the other model organisms. For example, while the longest UR length in *S. aureus* was 2591 in the Ensembl annotation, this increased to 12 332 in the Prodigal annotation. However, this was not consistent across all model organisms. Worth noting is how the longest UR in *P. fluorescens* decreased from 20 088 to 4264 for Ensembl and Prodigal annotations respectively.

**Table 4.** The number of clusters which have sequences from multiple genera

Cluster Type	1 Genus	2 Genera	3 Genera	4 Genera	5 Genera	6 Genera	>6 Genera
Ensembl-Only	2 296 340	203 993	19 566	4861	2036	990	2388
Ensembl-StORF	0	0	177	64	35	120	44
StORF-Combined-Ensembl	0	0	0	0	1	2	5
StORF	98 671	2573	199	63	27	25	27
StORF-Only	1 328 805	70 111	3483	964	359	244	368

The five cluster types are; (1) Ensembl-Only, (2) Ensembl-StORF, which are the clusters which have been extended into their respective genera group by the addition of StORF sequences, (3) StORF-Combined-Ensembl, which reports the number of gene families where StORF sequences combined at least 2 or more Ensembl cluster representatives together, (4) StORF, which are the same clusters as Ensembl-StORF but are counted only by their number of StORF sequences and (5) StORF-Only, which are the clusters which only contain StORF sequences and thus did not cluster with any Ensembl sequence. StORF-Only clusters with a single sequence were not included in these results.

There were clearly a number of URs that were long enough to contain complete CDS genes extracted from both the annotations of Prodigal and Ensembl - these were studied further.

### StORF-Reporter recovers Ensembl CDS genes missed by Prodigal

An analysis of the StORFs extracted from the URs of the six model organism genomes was undertaken using the annotations from Prodigal. StORF-Reporter was able to recover Ensembl-annotated genes that were missed by Prodigal, and also found many other potential genes that had sequence similarity to the Swiss-Prot database or proteins already annotated in the model organism genomes.

The ORForise platform reported that Prodigal was able to identify the vast majority of Ensembl genes from each of the model organisms, except for *M. genitalium* (2). For each of the model organisms, StORF-Reporter identified a high number of StORFs, as can be seen in Supplementary Table 5. However, StORF-Reporter is impeded by the mispredictions produced by Prodigal. For each model organism, Prodigal reported a number of CDSs which either had no equivalent in the Ensembl annotation, were elongated versions of Ensembl genes or were too inaccurate to be classified as ‘detected’, according to the ORForise platform. This meant that StORF-Reporter was only able to search for StORFs in the non-vitiated regions of the genome. For each model organism, between 2 and 46 non-vitiated missed Ensembl genes were recovered by using StORF-Reporter after the Prodigal annotation.

DIAMOND blastp was used to compare StORFs recovered from each model organism against the Swiss-Prot database and the proteomes derived from each respective model organism (see Supplementary Table 6). There were StORFs that had a high sequence similarity to proteins in Swiss-Prot that were not present in either the Prodigal or Ensembl annotations, and were investigated further.

### StORF-Reporter finds complete CDS genes not present in Ensembl annotations

StORF-Reporter found many StORFs that represent potential genes in the URs of the curated Ensembl annotations for each of the six model organisms. A number of StORFs had high sequence similarity to known protein-coding genes in the Swiss-Prot protein database (see Table 2). Each of these StORFs has the potential to contain not only undiscovered genes but also historic gene fragments or other functional units waiting to be characterised. There were also a number of StORFs long enough to be genes that did not have a high level of sequence similarity to Swiss-Prot (see Supplementary Figure S4 for further details on StORF lengths with and without Swiss-Prot hits). This could indicate that these sequences are not yet present in the databases or are fragmented in the genome.

To study the possibility that StORFs may capture instances of gene-duplication, for each of the model organisms, the reported StORFs were aligned to the proteomes of their corresponding genome. Between 6 and 73 StORFs were observed to contain an alignment to putative duplicate proteins from within the same genome (referred to as intra-genome hits). These StORFs are likely to represent duplicate genes that have not been detected by current genome annotation methods or have undergone some level of deleterious mutation. While we observed 58 intra-genome StORFs ‘hits’ in *M. genitalium*, it was the only model organism not to contain a StORF with an alignment of ≥80% of an Ensembl sequence. This is likely due to the ‘UGA/TGA’ codon repurposing and thus truncation and these results are reported in Table 2.

Additionally, we conducted RNA-Seq analysis for *M. genitalium* G7 using existing RNA-Seq data from the Centre for Genomic Regulation at https://www.ncbi.nlm.nih.gov/sra/ERX2249950 and found that StORFs are well supported by their transcripts. We mapped their cDNA reads to the genome using Bowtie2 (47) and counted the transcripts per million (TPM) supporting the Ensembl and StORF-Reporter annotated sequences using featureCounts (48) and edgeR (49). The results can be seen in Supplementary Figure S5. Many StORFs had higher TPM levels than the Ensembl annotation CDS genes. We removed 3 StORFs from this figure as they had extremely high TPM, and were clear outliers. One of these had a TPM of 2 504 698. This is nearly 100-fold higher than the other annotated genes. It had a partial match to a 16S rRNA sequence. Presumably, this is a now-defunct copy of the functional 16S in the genome.

We also investigated StORF-Reporter’s potential to identify known short proteins. Fuchs *et al.* (50) used proteogenomics and mass spectrometry to find novel short proteins in *S. aureus*. They found 24 ‘novel’ short proteins. However, only 5 of these 24 proteins were not already present in the Ensembl annotations. We compared these 5 (using DIAMOND blastp) to the StORFs and Prodigal predicted genes found for *S. aureus*. While Prodigal did not report any of these 5 proteins, StORF-Finder reported 3 that aligned with 100% identity (see Supplementary Figure S6). Using miniprot (51), we determined that the two other proteins were not present anywhere in our *S. aureus* genome.

Lastly, although we do not anticipate StORF-Reporter to return ncRNAs, due to their expected prevalence in URs and the default search parameters of StORF-Reporter being within the same regions, there is a likelihood of encountering overlaps. Therefore, to investigate what other genomic features other than CDS genes that StORFs may be capturing, we conducted a broad-scale search of all StORFs identified in the Rfam database (52) of non-coding and structural RNA genes. While Ensembl Bacteria v55 still used an outdated version of Rfam (v12.2), version 14.9 of Rfam is available online. We used this to find if there are Rfam families in the URs and StORFs reported for the 5109 genomes studied. 6.18% of the URs and 3.73% of the StORFs had Rfam hits using BLAST (53) (default settings). Additionally, for the 6 model organisms, while recognising the higher likelihood to have ncRNAs annotated, an average of around 7% URs and 7% StORFs had Rfam hits. While taking into account the errors and limitations inherent to both BLAST and the Rfam database, these low numbers are another piece of evidence supporting the StORF methodology.

### Extending the *E. coli* pangenome

StORF-Reporter found StORFs that both extend the core-gene set and create novel core-gene clusters potentially expanding our understanding of the *E. coli* pangenome.

169 *E. coli* genomes were extracted from the collection of 5109 Ensembl Bacterial genomes. From the 884 405 Ensembl protein sequences annotated in these genomes, 66 484 gene-clusters were formed, of which 28 677 were non-singletons. A median number of 3298 URs and 3345 StORFs were identified from the 169 genomes for a total of 563 263 URs and 579 661 StORFs (see Supplementary Table 7 for more detail). Representatives from each of the Ensembl-Only clusters (all 66 484-including singletons) were combined with the 579 661 StORFs identified from the 169 *E. coli* genomes and the same CD-HIT clustering methodology was applied. This resulted in a total of 139 570 clusters, of which from a total of 32 049 non-singletons, 24 835 contained only StORF sequences.

The clustering of the Ensembl proteins resulted in 752 core-gene families. Most interestingly, with the addition of the StORF sequences, many Ensembl accessory clusters were extended into the core, soft-core and accessory (#66, #89 and #568 respectively) thresholds (see Table 3 for further detail). These constitute gene families that are likely to have been left out of many studies due to them incorrectly being classified as not part of the core. Additionally, there were clusters that contained both Ensembl and StORF sequences classified as core-gene families on the basis of their StORF sequences alone. While they were grouped with Ensembl genes, the Ensembl genes did not contribute to the distribution of the cluster (2 were core, 21 were soft-core and 587 were accessory). Moreover, there were 127 and 359 StORF-Only clusters which formed core and soft-core gene families, respectively. These clusters constitute entirely novel core and soft-core gene families missing from the Ensembl annotations.

Supplementary Figure S7 shows the distribution of the unique *E. coli* genomes present in the Ensembl-Only, Ensembl-StORF and StORF-Only clusters all followed a similar U-shaped curve. Notably, the size of the clusters that were often extended by the StORFs was often increased by a substantial amount. For example, the proportion of clusters containing Ensembl sequences with 10 or fewer unique genomes was reduced from around 70% to 50% with the addition of the StORF sequences. Only 162 of the 5260 Ensembl gene families that clustered with StORF sequences did not extend into additional *E. coli* genomes.

The inherently limiting process of using hard-coded sequence similarity and length cut-offs to distinguish gene families has inevitably brought forward a number of interesting cases. One example can be seen in the combining of multiple Ensembl protein representative sequences, and thus gene families, into a single new cluster by the addition of StORFs. This was a result of StORF sequences effectively bridging the gap between multiple Ensembl sequences (defined henceforth as StORF-Combined-Ensembl clusters).

**Figure 4.**
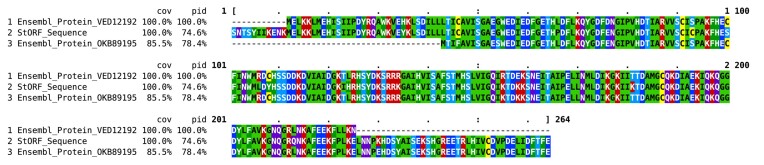
Clustal Omega multiple sequence alignment of the two Ensembl representative sequences, VED12192 and OKB89195, which were combined in *E. coli* pangenome cluster #13 287 by the additional StORF sequence. This combination is done because the StORF sequence extends passed VED12192 begins and to where OKB89195 ends. These two sequences on their own do not align together for long enough for the clustering parameters, thus the reason they originally formed independent clusters. Additionally, they were also annotated as different proteins by Ensembl.

The 214 aa representative protein VED12192 of Ensembl cluster #7787 (consisting of 14 sequences and spanning 13 strains, one of which contained a duplicate within the same genome), was clustered with the representative 222 aa protein OKB89195 of Ensembl cluster #9205 (consisting of 10 sequences from 10 strains) when StORF sequences were included in the second round of CD-HIT clustering. Previously, these two Ensembl proteins, reported as a ‘H repeat-associated protein’ (VED12192.1) and a ‘transposase’ (ANJ36103.1), respectively, were distinct enough to be clustered separately. However, they were both clustered together into combined cluster #13 287 with 3 StORFs from 3 additional strains. The 264 amino acid representative StORF sequence from this cluster was reported with multiple transposase hits, including ‘ISAs1 family transposase’ (WP_194174573.1) and ‘H repeat-associated protein’ (STK57581.1) in GenBank (54). The representative StORF in this cluster is longer than the Ensembl genes (around 40-50 aa), and as such, CD-HIT chose this StORF to be its representative sequence for the combined cluster. The sections of the StORF sequence that aligns to only one of the Ensembl proteins is highly similar and suggest that the StORF sequence could either be another allele of this gene family or an instance of alternative start and/or stop codon use. As can be seen in the Clustal Omega (55) multiple sequence alignment of the representative sequences of the above-mentioned clusters in Figure 4, their sequence identity was high, $\ge 90\%$ (the minimum CD-HIT percentage identity for clustering) along the regions in which they aligned.

A further example of this type of clustering can be seen in Supplementary Figure S8 which reports a multiple sequence alignment of three Ensembl representative sequences (PEH97659, QCH93547 and AWN77897) combined into a new cluster with a single representative StORF sequence (Ensembl-StORF cluster #3785). As we can observe from their alignment, they are very similar and are further examples of the limitations we impose when attempting to represent natural elements in ‘computationally-friendly’ categories. There were 87 of these StORF-Combined-Ensembl clusters which together had clustered 183 Ensembl representative sequences, and thus their clusters, together. These clusters are presented separately in Table 3.

Lastly, a large number of singleton Ensembl gene families have been extended into additional *E. coli* genomes with StORF sequences. For example, 337 Ensembl singleton gene families were extended into at least additional 10 genomes and at least one was extended into the core pangenome. This singleton Ensembl-Only cluster (Ensembl-Only cluster #44 706) consisted of a single 52 amino acid *E. coli* Ensembl gene (reported in GenBank (54) as a hypothetical protein (ANK02814.1)). When combined with the StORF sequences, this sequence was clustered into the core pangenome as combined Ensembl-StORF cluster #64.

### StORFs identified within and across multiple genera

Previous analyses of URs and StORFs have been conducted on the same species. It could, therefore, be assumed that some StORFs (and of course some Ensembl genes) that have been identified are remnants from a multitude of different genomic factors such as assembly error, genomic structural elements, or additional processes we are yet to fully understand. However, in this study, a large number of StORFs were identified (with and without similarity to Ensembl genes) across multiple genera, forming both novel and extended cross-genera gene families.

The complete collection of 20 153 937 Ensembl-annotated protein sequences was collected from the 5109 Ensembl Bacteria genomes (see Supplementary Table 1 for details on the genomes used). A median number of 2589 and 2432 URs and StORFs were identified for each of the 5109 genomes for a total of 13 656 918 URs and 13 197 690 StORFs (see Supplementary Table 8 for more detail). Using CD-HIT, Ensembl-Only, Ensembl-StORF, and StORF-Only clusters were identified that span multiple genera. The distribution of Ensembl-Only clusters is broad, and many clusters are formed from more than ten different genera (see Table 4). Furthermore, not only have Ensembl-Only clusters been added to and extended by StORFs (creating Ensembl-StORF clusters), but also substantial numbers of StORF-Only clusters have been found with StORFs from multiple genera. Many clusters were identified with Ensembl and StORF sequences from the same genera and genomes and are likely to represent important candidates for gene duplication studies and should be added to canonical annotations.

StORFs have changed the diversity and distribution of gene families by extending the canonical Ensembl gene clusters into additional genera. One example of this can be found in the Ensembl-Only cluster #897 563 that consisted of 4 exactly the same 30 amino acid Ensembl genes from two different *Klebsiella pneumoniae* genomes (reported in GenBank) as a hypothetical protein). The representative sequence, when combined with the StORF sequences of all 5109 Ensembl genomes, was clustered into a comparatively large Ensembl-StORF combined cluster (cluster #819), with StORFs spanning multiple different genera. This cluster consisted of 158 StORF sequences from a total of 30 genomes and 14 genera (*Escherichia, Citrobacter, Enterobacter, Bacillus, Aeromonas, Providencia, Acinetobacter, Cronobacter, Enterobacteriaceae, Haemophilus, Mycoplasma, Salmonella, Klebsiella, Neisseria* and *Klebsiella*).

As another example, the singleton Ensembl cluster #4 211 958, which consisted of a single short 38 aa sequence in a *Salmonella enterica* genome, was greatly extended by 68 StORF sequences from 10 additional genera (*Achromobacter, Acinetobacter, Citrobacter, Corynebacterium, Cronobacter, Enterobacter, Enterobacteriaceae, Escherichia, Klebsiella* and *Pseudomonas*), as part of the Ensembl-StORF combined cluster #2865. This protein is reported as a ‘replication initiator protein’ in GenBank (AXH26447.1) with a 100% sequence identity match and is another example of StORF-Reporter identifying putative short-proteins across a range of genera and species.

As with the *E. coli* pangenome study, the hard-coded cut-offs used for the CD-HIT gene family analysis produced a number of clusters which combined multiple Ensembl representative sequences which were previously clustered separately. There were 799 StORF-Combined-Ensembl clusters that together combined 1702 Ensembl-Only clusters. Additionally, many of the StORF sequences were found in genera that were not present in the original and separate Ensembl-Only clusters. An example of one such cluster, Ensembl-StORF cluster #26 643, combined 12 Ensembl-Only representative proteins together with 3 StORF sequences. These 12 clusters, all annotated as involved in carbon storage regulation, ranged from singletons to large gene families spanning over 200 proteins from 20 genera. This new cluster consists of 770 Ensembl proteins and 3 StORF sequences which makes it the second largest cluster by sequence count. Supplementary Figure S9 shows the phylogenetic tree built with ClustalO and FastTree (56) and plotted with iTOL (57) (see Supplementary Figure S10 for the multiple sequence alignment). This phylogenetic tree reports how along with the Ensembl representative protein sequences, the StORF sequences are positioned according to their taxa. Additionally, the multiple sequence alignment reports how for some amino acid positions, StORF sequences can be more similar to one or more Ensembl genes as than other Ensembl sequences. Therefore, it could be argued that as these StORF sequences bridge the gap between the 12 separate Ensembl gene families, they have changed the dynamics of these inter-genera gene families. These type of clusters were included in the results of Table 4, separately in row ‘StORF-Combined-Ensembl’.

StORF-Reporter was also able to identify a number of clusters containing only StORF sequences that spanned different genera. One example of this can be seen in the StORF-Only cluster #2618 which spans 6 genera and consists of 75 genomes and sequences. At 191 amino acids long, the representative StORF sequence of this cluster had a 98.5% identity and 80% query coverage hit to a ‘Small heat shock protein’ (AHM76795.1) sequence in GenBank.

## Discussion

### The unannotated regions of prokaryote genomes contain many potential genes

The high gene densities observed in canonical prokaryotic annotations have long been “perceived as evidence of adaptive genome streamlining” (1). However, this is in contradiction with the observations that many prokaryote genomes contain a large number of long URs that comprise between 10 and 20% of their genomes (see Table 2 and Supplementary Tables 3 and 4). Despite advances in gene discovery, proteogenomics, and integrating diverse omics data (11,58–62), there is still scope to improve genome annotation strategies. Previous findings (2) confirmed that contemporary methods such as Prodigal (30) accurately detect the majority of CDS genes (in genomes which use the ‘universal’ codon table). However, as shown in this work, most genomes exhibited thousands of URs that contain genes missed by state-of-the-art gene prediction methods and are thus missing from the canonical Ensembl annotations or exhibited high levels of sequence identity to proteins in the Swiss-Prot database.

It is difficult to know which proportion of the StORFs identified in this study are a result of the current limitations of genome annotation methods or are in fact genes that are no longer expressed, possibly due to mutations in a promoter region (putative pseudogenes). This is a problem that is not likely to be solved soon, as evidenced by the large numbers of ‘hypothetical’ or ‘putative’ genes that exist in canonical genome annotations, even in model organisms such as *E. coli* (63). Interestingly, the stop codon abundances and usages presented in Supplementary Tables 9 and 10, suggest that StORFs without homology to known CDS genes may be at least partially validated with stop codon usage analysis. Furthermore, since the coding and non-coding regions of prokaryote genomes are believed to evolve separately, reacting to intra- and inter-genomic pressures independently (64), mutation rate studies could also potentially be used as evidence that they are functional genes. In addition, the observation that many of the StORFs appear to be gene duplicates and/or members of broad gene families validates that our approach can identify real genes (see ‘Intra-Genome’ results in Table 2). Regardless of whether these duplicates are pseudogenes, a type of gene that is too often ignored, their presence is important and their addition to prokaryotic annotations is likely to enhance our understanding of how these genomes evolve. For example, we have identified large numbers of StORF-Only clusters, those gene families consisting only of StORF sequences that did not have a high level of similarity to any known gene, spanning over 10 genera in some cases. These may represent CDS sequences or cryptic genomic elements, previously overlooked, and are prime targets for future investigations.

### The ‘Stop-Open Reading Frame’ is a valuable and unambiguous concept

As previously mentioned, our definition of a StORF is synonymous with the Sequence Ontology (14) definition of an ORF—“The in-frame interval between the stop codons of a reading frame”. This differs from the canonical use of the term ‘ORF’ (a coding frame beginning with a start codon and ending with a stop codon) which has become the standard approach for most genome annotation tools (2). Unlike the use of ‘ORF’ in genome annotation, StORFs have an unambiguous description and use. Additionally, StORFs offer unbiased identification of genes, even those with non-canonical start codon profiles. Lastly, as StORFs are reported from URs already identified in previously annotated genomes, we are building upon the excellent work of previous researchers, rather than restarting annotations from scratch. The StORF-Adder tool is provided with the ORForise package (2) to supplement existing genome annotations from Ensembl or workflows such as Prokka and Bakta, retaining the same output format. This allows downstream analysis tools such as Roary (65), Panaroo (66) and Coinfinder (67) to interpret these new annotations without further intervention.

### StORFs can extend pangenomes

For some time, it has been known that a single genome is not enough to characterise the functional profile of a species, instead requiring a pangenomic approach. As pangenomes consist of a species-wide collection of core-genes that are present in most/all genomes and accessory genes that are only present in some genomes, missing annotations will undoubtedly impact our understanding of species-wide gene families. Indeed, while our pangenomic study of *E. coli* has shed light on the potential for yet more diversity in its accessory gene collection, it is the increased set of core and soft-core genes, both expanded and novel, that truly showcase the advantage of StORF-Reporter. These gene families are candidates for studying how a species can adapt to a changing environment (68) as they have been retained despite the pressures from genome streamlining (69–71) and are thus more likely to be functionally important. Fundamentally, while the identification of these StORFs has provided additional genomic knowledge previously left in the dark corners of the URs of their genomes, to truly use the nascent information they may harbour, more experimental work is needed.

### StORFs can extend intra- and inter-genera gene collections

The results of the StORFs reported in the six model organisms and in the *E. coli* pangenome have shown wide levels of diversity and spread. However, it could be possible that many of these StORFs are artefacts of genome assembly or are species-specific structural elements and not primarily CDS genes. Therefore, the inter-genera analysis of the 5109 genomes from Ensembl Bacteria was key not only to study the presence of StORFs in other diverse species, but also to provide evidence to further validate them as putative CDS genes.

StORFs were found in many different genera with high sequence similarities (≥90%) (see Table 4). As in the *E. coli* pangenomic study, many StORFs from the same cluster were found in different locations in various genomes, or in the case of duplication, in different regions, and thus URs of the same genome. The presence of these StORFs, adds credence to the hypothesis that they are not structural elements or other genomic artefacts but instead functional (past or present) CDS genes.

As StORF-Reporter is species- and start codon-agnostic, we were better able to observe the full spectrum of variation within a gene family. This provides yet further justification for the need for species-agnostic gene annotation methodologies which do not rely on established model organisms or databases.

### Are some StORFs pseudogenised genes?

The potential diversity of all possible protein sequences is vast (72). A typical protein length of about 300 amino acids can have theoretically up to 20^300^ different possible polypeptide chains, yet current protein databases only contain a tiny fraction of this, such as Swiss-Prot, which contains a little over 500 000 protein sequences. Many of these are duplicates or very similar to each other and a considerable number of these are not experimentally validated. Adding to this complexity is the vast number of pseudogenes, which were likely functional proteins in the recent past, identified through the extensive genome sequencing of the last few decades. While mutation rates have long been studied in prokaryotes (73,74), there are still unresolved questions surrounding the speed at which pseudogenes are formed. Some studies have shown that pseudogenes are more likely to be a result of failed horizontal gene transfer (75). Furthermore, while archaea and most non-pathogenic bacteria exhibit greater retention of ancient gene remnants, obligate pathogenic bacteria tend to have younger pools of pseudogenes (75). As such, the types of genes pseudogenised and the rate at which they are mutated are highly variable between species and are likely to be related to environmental-specific cues.

Unfortunately, current methods fail to address the prevalence of gene pseudogenisation; therefore, the true extent of gene pseudogenisation is likely to remain unclear for the foreseeable future. As reported by Goodhead *et al.* (76), of the five different processes causing a gene to be pseudogenised (premature stop, loss/change of function domain, loss of start codon, loss of promoter/RBS, frameshift), a StORF has the potential to identify all of them (some in fragmentary form). Additionally, while a single StORF could only report one fragment of a gene pseudogenised by a premature stop codon, the combination of multiple consecutive StORFs in the same reading frame has the potential to recover the entire sequence of a pseudogenised gene. urthermore, as a StORF captures a portion of the 5’ untranslated region of a CDS gene, it may also identify gene silencing mutations, such as start codon or ribosomal binding sites.

## Conclusion

Genome annotation is a mature but continually developing field. With each new sequencing project, the proportion of uncharacterised sequences found can vary significantly, and often hundreds of novel genes are discovered with varying similarity to those from previously sequenced taxa (77), suggesting that considerable gene diversity remains to be discovered. Historical and systematic biases and errors mean that some types of genes will almost always be missed in these studies, resulting in many tools reporting the same incorrect intergenic regions (2). This might lead researchers to feel a sense of false security when using canonical annotation tools, but, as we have demonstrated, the StORF-Reporter methodology can enhance current annotations, reporting many missed genes in these regions.

One major limiting factor shared across many if not all genome annotation analysis studies is the use of a genomic database such as Ensembl Bacteria as ground truth (under the acceptance that they represent a near-complete reference). In this study, we tested that assumption of completeness. We used StORF-Reporter to investigate the URs extracted from over 5000 Ensembl annotated genomes. StORF-Reporter was able to identify more than 480 StORF-Only core and soft-core gene families from the *E. coli* pangenome, which were distinct from any of those present in Ensembl. This constitutes more than a 17% increase in the size of the core and soft-core gene collection for the *E. coli* pangenome. Moreover, a large number of StORFs were highly similar to known proteins present across multiple genera. Both sets of StORFs showcase how even highly-conserved gene families are still missing from canonical annotations. This is strong evidence that StORF-Reporter is able to recover novel functional sequences even when applied to well-studied reference genomes from model organisms.

The URs and StORFs identified by StORF-Reporter can be interpreted and used in a number of different ways. Once StORFs have been recovered, the remaining regions of DNA in the collection of URs for any genome are not without significance. StORF-Reporter makes it easier to study these cryptic regions, which could be classified as the ‘real unknown-unknowns’. We provide a tool for the extraction of these unknown-unknowns in the StORF-Reporter package.

In summary, genomes are not static; elements are added, removed, and mutated over time, and thus the content of a genome is dynamic in nature. This work represents a movement toward the thesis that every nucleotide in the genome should be labelled, regardless of whether it belongs to a functional gene, a pseudogene, a gene fragment that is no longer functional, or any other genomic feature. Therefore, we encourage the community to use StORF-Reporter to enhance both canonical and novel genome annotations.

## Supplementary Material

gkad814_supplemental_file

## Data Availability

The StORF-Reporter package is available on (https://doi.org/10.5281/zenodo.8408339) and as a PyPi package (https://pypi.org/project/StORF-Reporter/). The full ORForise package which has been updated with the StORForise sub-package is also available at (https://doi.org/10.5281/zenodo.8408339) and as a PyPi package (https://pypi.org/project/ORForise/). The StORF-Reporter source code, extracted URs and StORFs from the entire collection of 5109 Ensembl genomes are available at figshare (https://doi.org/10.6084/m9.figshare.23257871).
