# Supplementary material for: StORF-Reporter: finding genes between genes
Source: Nucleic Acids Res. 2023 Oct 28;51(21):11504–17. doi: 10.1093/nar/gkad814 (PMC10682499; doi:10.1093/nar/gkad814)
Supplement: gkad814_supplemental_file [file gkad814_supplemental_file.pdf]

Supplementary Information
StORF-Reporter: Finding Genes between Genes

Nicholas J. Dimonaco [1,2,3,4,5*], Amanda Clare [2], Kim Kenobi [6], Wayne Aubrey [2]†, and Christopher J. Creevey [5]†

[1]Institute of Biological, Environmental and Rural Sciences, Aberystwyth University, Aberystwyth, SY23 3PD, Wales, UK
[2]Department of Computer Science, Aberystwyth University, Aberystwyth, SY23 3DB, Wales, UK
[3]Department of Medicine, McMaster University, Hamilton, ON, Canada
[4]Farncombe Family Digestive Health Research Institute, McMaster University, Hamilton, ON, Canada
[5]School of Biological Sciences, Queen's University Belfast, Belfast, BT7 1NN, Northern Ireland, UK
[6]Department of Mathematics, Aberystwyth University, Aberystwyth, SY23 3BZ, Wales, UK

*To whom correspondence should be addressed. *nicholas@dimonaco.co.uk*
†The authors wish it to be known that, in their opinion, the last two authors should be regarded as Joint Senior Authors.

1 Supplementary Figures

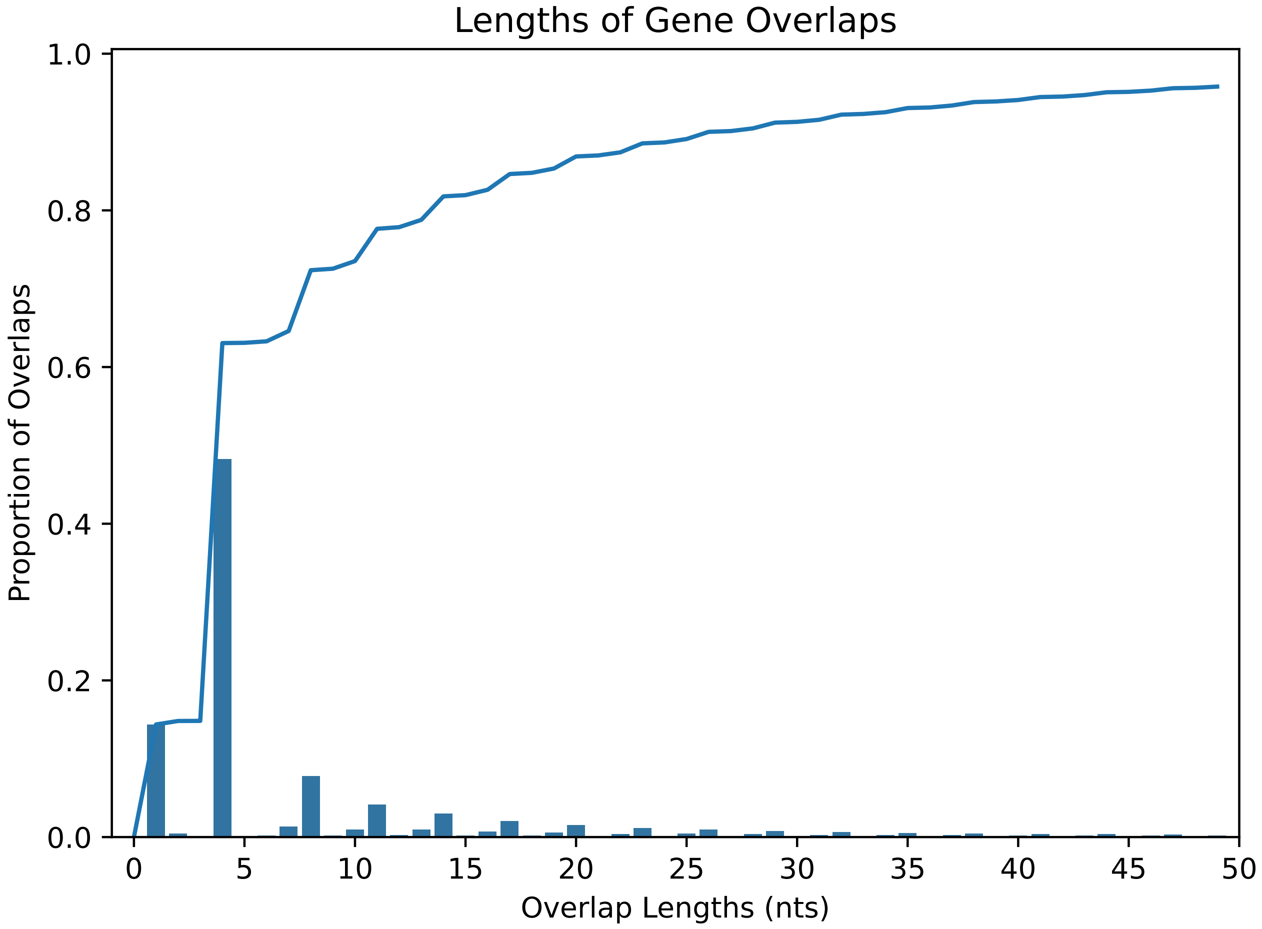

Supplementary Figure 1: Shown here are the proportion of overlap lengths in nucleotides between all CDS genes from the 5,109 filtered genomes from Ensembl Bacteria. The blue line reports the cumulative proportion of gene overlaps increasing very little after 5-10 nt.

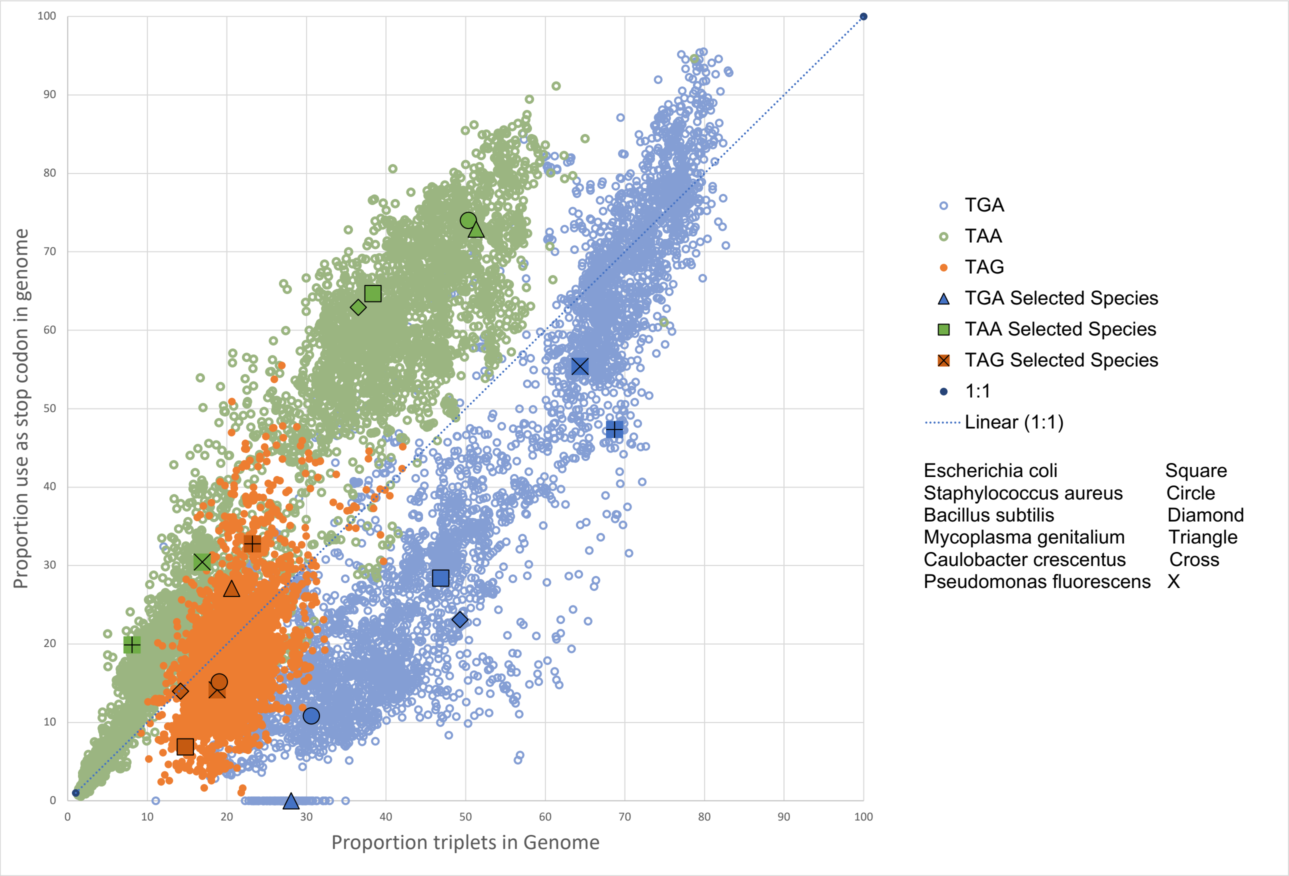

Supplementary Figure 2: Graph showing stop codon usage (y axis) and proportion of triplets (x axis) in the 5,109 genomes from Ensembl Bacteria. For each of the 6 model organisms, this show the distribution across the entire genome of the three triplets TAA, TAG and TGA in all six reading frames and stop codon usage (i.e. the actual relative usage in Ensembl CDS genes of the three different stop codons).

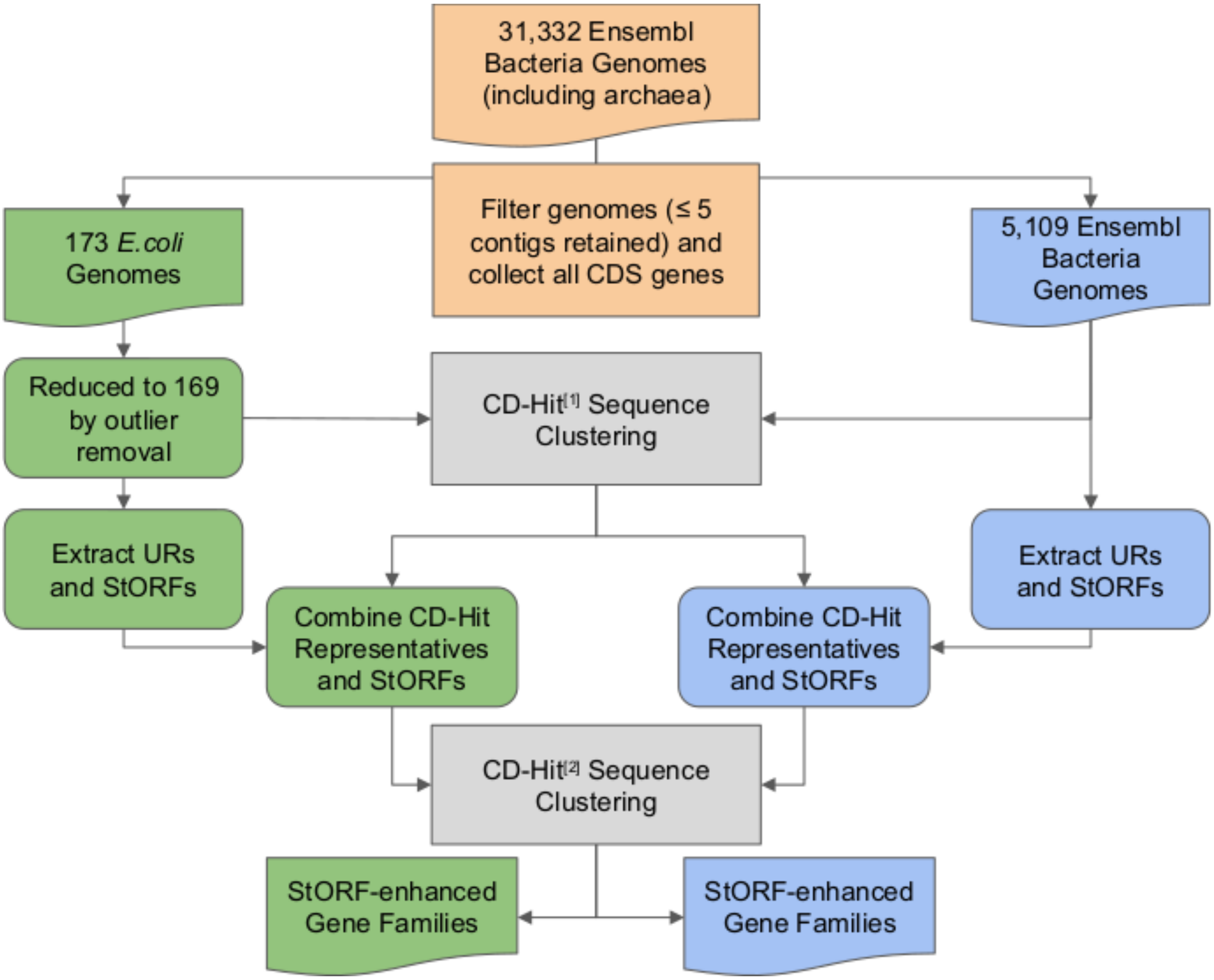

Supplementary Figure 3: This workflow diagram presents the process used to create the *E. coli* pangenomic and cross-genera gene families. The same original Ensembl input data and genome filtering was used for both studies (orange boxes) and the same CD-Hit clustering protocol was undertaken (grey boxes). The green boxes indicate the specific route the *E. coli* genomes took and the blue boxes report the same for the cross-genera study. There are two separate CD-Hit stages which are applied the same to both datasets and are described as: [1] This CD-Hit clustering stage was performed only on the amino acid sequences reported in the Ensembl Bacteria annotations, [2] This CD-Hit clustering stage was performed on the Ensembl amino acid sequence representatives reported by the previous CD-Hit analysis and the StORFs identified from the Ensembl annotations. The same parameters of 90% sequence identity and shorter sequence length cut offs were applied to both.

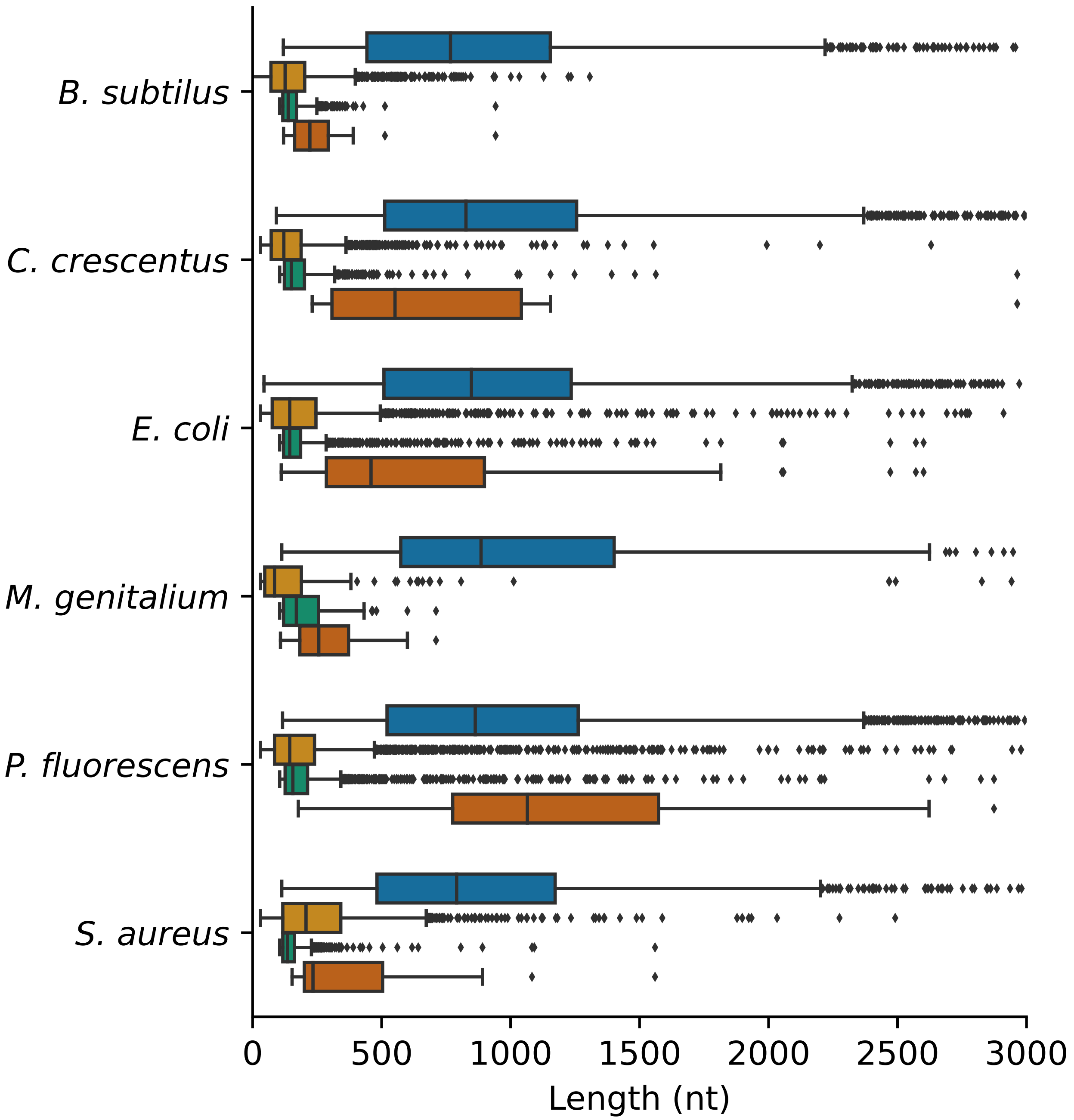

Supplementary Figure 4: Reported here are the nucleotide lengths of the Ensembl annotated genes for each of the six model organisms (blue), the unannotated regions (URs) (light orange), the StORFs (Stop-ORFs) identified from the URs (green) and the StORFs which had a high sequence similarity to known protein coding genes in Swiss-Prot (>=60% bitscore) (dark orange). X axis truncated at 3,000 nt.

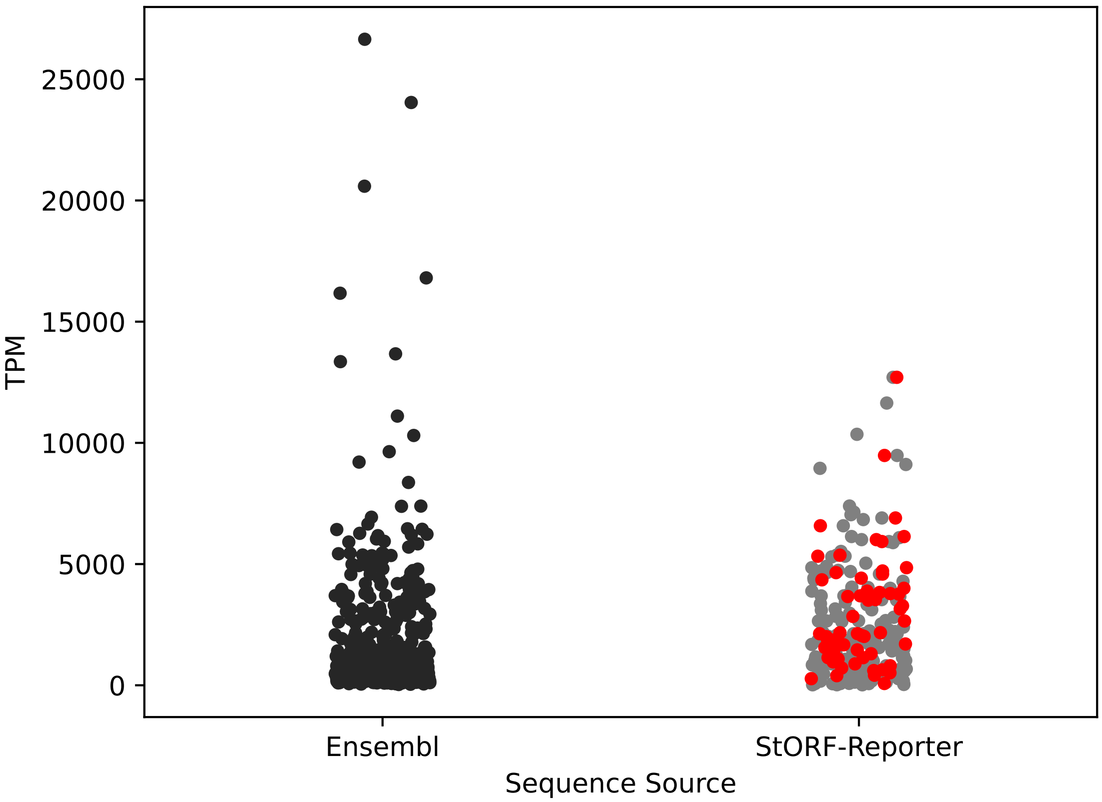

Supplementary Figure 5: Strip plot of TPM (transcripts per million) for Ensembl (Black) and StORF-Reporter (Grey and Red) annotated sequences from *Mycoplasma genitalium* with the Y axis truncated at 25,000. While one Ensembl CDS gene had a TPM higher than 25,000 (26,649), three StORFs were reported with a nearly 100-fold higher TPM than the average. StORF points are coloured red if they have sequence similarity using BLAST default parameters to Ensembl annotated genes. These are likely to be paralogs or fragments left by a duplication event that are now found by StORF-Reporter.

```
                      cov     pid   1 [        .         .         .] 31
1 StORF_Sequence   100.0% 100.0%      TSYLKGAIHMEFVAKLFKFFKDLLGKFLGNN
2 Short_Protein_4   71.0% 100.0%      ---------MEFVAKLFKFFKDLLGKFLGNN

                      cov     pid   1 [        .         .         .         .       ] 49
1 StORF_Sequence   100.0% 100.0%      FVRYREMRYVTMRQFIKRTVKTILVGYVIKFIRNKLSGKSSHPTDNKHN
2 Short_Protein_7   87.8% 100.0%      ------MRYVTMRQFIKRTVKTILVGYVIKFIRNKLSGKSSHPTDNKHN

                       cov     pid   1 [        .         .         .         .         :         .         .       ] 78
1 StORF_Sequence    100.0% 100.0%      HLFTRLQYFEVIKEVSRILTFSEQNALAKIDDLMNTYCNQCPIKTRLRKLEGKTKAHHFCINECSIGKEIKQLGNELQ
2 Short_Protein_16   80.8% 100.0%      ---------------SRILTFSEQNALAKIDDLMNTYCNQCPIKTRLRKLEGKTKAHHFCINECSIGKEIKQLGNELQ
```

Supplementary Figure 6: Reported here are three multiple sequence alignments of a subset of the 24 short-proteins experimentally validated in *S. aureus* which were identified by StORF-Reporter but not Prodigal or Ensembl. All three were found by StORF-Reporter with default settings with a short upstream non-coding segment.

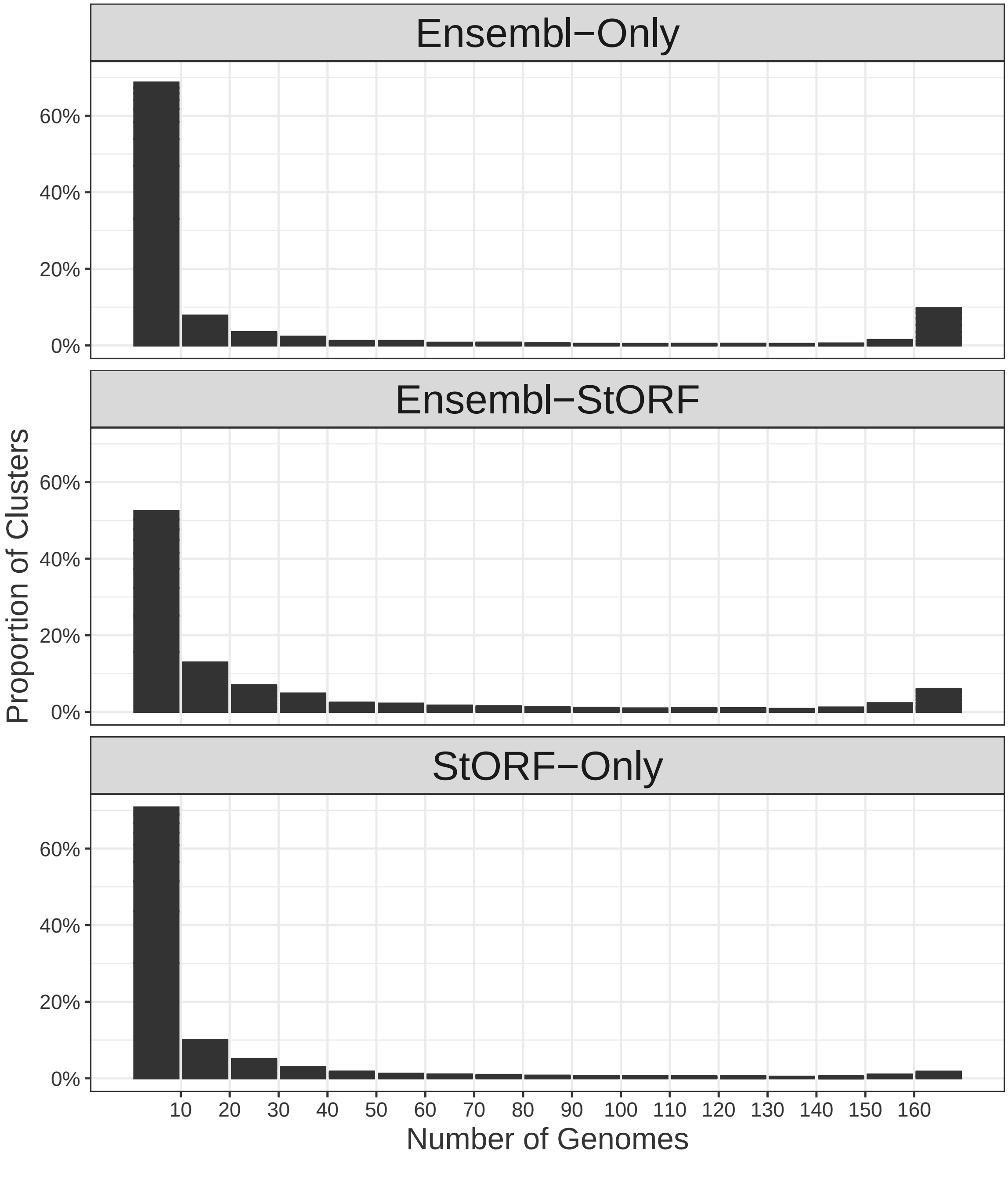

Supplementary Figure 7: The distributions of gene families across the 169 *E. coli* pangenome for the Ensembl-Only, Ensembl-StORF and StORF-Only clusters. The U-shaped curve is consistent throughout the three cluster types with Ensembl-StORF containing slightly larger gene family clusters as expected due to the added StORF sequences as compared to Ensembl-Only. While the distribution is more towards the lower end for StORF-Only, a similar, albeit much less pronounced U-shaped curve is observed.

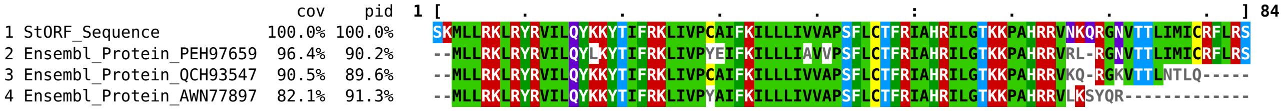

Supplementary Figure 8: ClustalO multiple sequence alignment of the three originally independent Ensembl representatives sequences, PEH97659, QCH93547 and AWN77897 which were clustered together by the addition of StORF sequences which formed the single *E. coli* pangenome cluster 3,785. These three Ensembl representative sequences and their clusters have been grouped together by 33 StORF sequences, thus completely changing the dynamics of this set of gene families. The sequences did not cluster together in the first round of clustering because their percentage identity was less than 90% from each other.

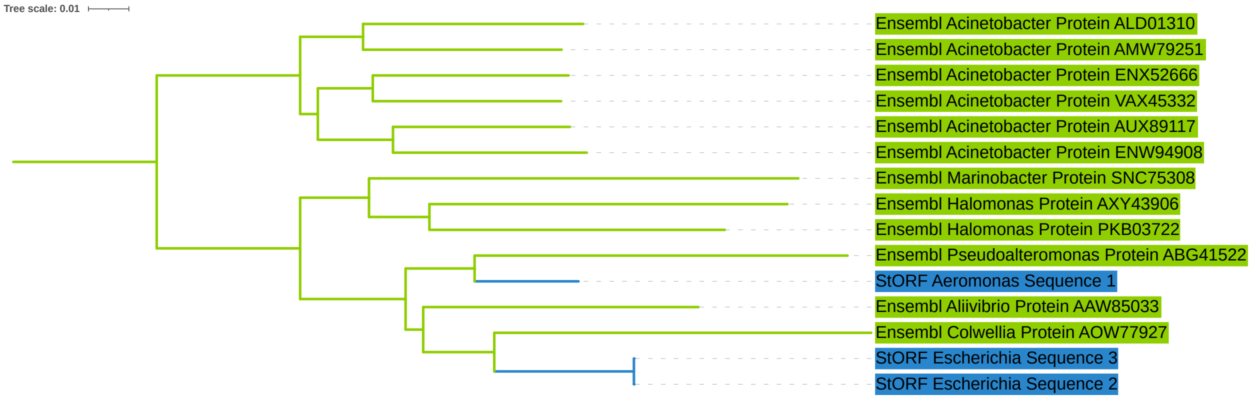

Supplementary Figure 9: This is a phylogenetic tree built from the amino acid sequences of combined Cluster 26,643. This cluster consists of 12 Ensembl cluster representatives and the 3 StORF sequences which clustered to those representatives. This tree was created using ClustalO and FastTree and was midpoint rooted.

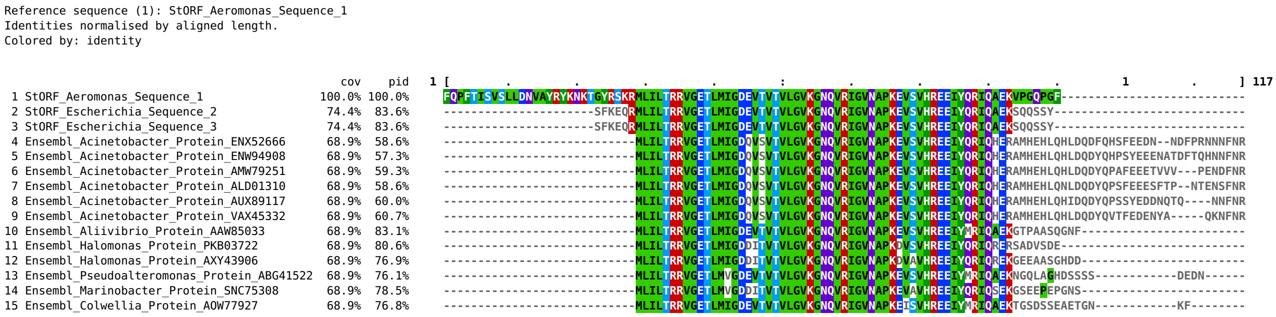

Supplementary Figure 10: ClustalO multiple sequence alignment from the amino acid sequences of combined Cluster 26,643. This cluster consists of 12 Ensembl cluster representatives and 3 StORF sequences. As can be seen in this alignment, for some amino acid positions, StORF sequences can be more similar to one or more Ensembl genes as than other Ensembl sequences.

2 Supplementary Tables

Acetobacter	13	Clostridium	81	Leuconostoc	6	Propionibacterium	18
Achromobacter	10	Collimonas	8	Limnohabitans	5	Proteus	6
Acidipropionibacterium	9	Colwellia	7	Listeria	22	Providencia	10
Acidithiobacillus	5	Comamonas	9	Lysinibacillus	9	Pseudoalteromonas	24
Acidovorax	23	Corynebacterium	81	Lysobacter	11	Pseudomonas	276
Acinetobacter	82	Cronobacter	7	Mannheimia	6	Pseudonocardia	8
Actinobacillus	11	Cupriavidus	16	Marinobacter	17	Psychrobacter	12
Actinomyces	17	Cutibacterium	17	Massilia	10	Pyrobaculum	8
Actinoplanes	7	Dehalococcoides	6	Mesoplasma	12	Pyrococcus	8
Aerococcus	6	Deinococcus	16	Mesorhizobium	23	Ralstonia	18
Aeromicrobium	10	Delftia	6	Metallosphaera	10	Rathayibacter	5
Aeromonas	28	Desulfitobacterium	5	Methanobacterium	11	Rhizobium	35
Afipia	6	Desulfotomaculum	5	Methanobrevibacter	7	Rhodobacter	7
Agrobacterium	17	Desulfovibrio	18	Methanocaldococcus	6	Rhodobacteraceae	5
Agromyces	7	Devosia	5	Methanococcus	9	Rhodococcus	44
Akkermansia	8	Dickeya	10	Methanosarcina	27	Rhodopseudomonas	8
Alcanivorax	5	Edwardsiella	6	Methanothermobacter	6	Rickettsia	33
Aliivibrio	5	Ehrlichia	7	Methylobacterium	14	Rothia	7
Altererythrobacter	10	Elizabethkingia	7	Methylorubrum	9	Ruegeria	7
Alteromonas	16	Enterobacter	24	Microbacterium	38	Ruminococcus	9
Amycolatopsis	13	Enterobacteriaceae	6	Micrococcus	8	Saccharolobus	12
Anoxybacillus	6	Enterococcus	48	Microcystis	7	Saccharomonospora	7
Archaeoglobus	5	Entomoplasma	6	Micromonospora	43	Salmonella	43
Arcobacter	12	Erwinia	9	Moraxella	15	Selenomonas	7
Arthrobacter	22	Erythrobacter	13	Mucilaginibacter	11	Serratia	34
Avibacterium	5	Escherichia	178	Muricauda	5	Shewanella	34
Azoarcus	8	Eubacterium	17	Mycobacterium	49	Shigella	14
Azospirillum	12	Exiguobacterium	5	Mycobacteroides	6	Sinorhizobium	14
Bacillus	207	Fibrobacter	5	Mycolicibacterium	24	Sorangium	5
Bacteroides	23	Flavobacteriaceae	8	Mycoplasma	82	Sphingobacterium	8
Bartonella	29	Flavobacterium	36	Myroides	6	Sphingobium	20
Bifidobacterium	55	Francisella	18	Myxococcus	6	Sphingomonas	31
Blautia	6	Frankia	5	Natrinema	5	Sphingopyxis	16
Bordetella	26	Fusobacterium	34	Neisseria	29	Spiroplasma	21
Borrelia	8	Gardnerella	7	Nitrosomonas	8	Staphylococcus	79
Borreliella	8	Geobacillus	20	Nocardia	15	Stenotrophomonas	21
Bosea	5	Geobacter	12	Nocardioides	12	Streptococcus	179
Brachybacterium	7	Gluconobacter	5	Nocardiopsis	6	Streptomyces	226
Brachyspira	11	Gordonia	10	Nonlabens	9	Sulfitobacter	8
Bradyrhizobium	30	Gramella	6	Nostoc	12	Sulfolobus	17
Brevibacillus	8	Haemophilus	20	Novosphingobium	7	Sulfurospirillum	5
Brevibacterium	13	Haloarcula	6	Oceanobacillus	5	Synechococcus	30
Brevundimonas	13	Halobacterium	6	Ochrobactrum	7	Tenacibaculum	8
Brucella	15	Haloferax	5	Paenibacillus	61	Tetrasphaera	5
Burkholderia	80	Halomonas	23	Pandoraea	9	Thermoanaerobacter	8
Caldicellulosiruptor	9	Haloquadratum	5	Pantoea	13	Thermococcus	30
Calothrix	9	Helicobacter	107	Paraburkholderia	29	Thermotoga	8
Campylobacter	52	Herbaspirillum	8	Paracoccus	14	Thermus	11
Candidatus	87	Hydrogenophaga	6	Pasteurella	12	Thioalkalivibrio	5
Capnocytophaga	20	Hymenobacter	10	Pectobacterium	6	Treponema	25
Carnobacterium	7	Hyphomicrobium	6	Pediococcus	9	Variovorax	9
Caulobacter	10	Janthinobacterium	16	Pedobacter	7	Veillonella	8
Cedecea	5	Kitasatospora	6	Peptoniphilus	5	Vibrio	53
Celeribacter	5	Klebsiella	59	Phaeobacter	7	Virgibacillus	5
Cellulomonas	7	Kocuria	8	Photobacterium	5	Weissella	8
Chitinophaga	6	Komagataeibacter	8	Planococcus	11	Wolbachia	7
Chlamydia	18	Lactobacillus	142	Plantibacter	5	Xanthomonas	41
Chlorobium	5	Lactococcus	23	Polaribacter	15	Xenorhabdus	8
Chromobacterium	9	Legionella	26	Polynucleobacter	8	Xylella	6
Chryseobacterium	37	Leifsonia	12	Porphyromonas	8	Yersinia	23
Citrobacter	27	Leptolyngbya	6	Prevotella	34	Zymomonas	5
Clavibacter	7	Leptospira	9	Prochlorococcus	16		

Supplementary Table 1: Listed here are the 5,109 genomes grouped into their 247 genera after filtering that were used in the cross genera study.

Escherichia_coli_1303_gca_000829985
Escherichia_coli_53638_gca_000167915
Escherichia_coli_536_gca_000013305
Escherichia_coli_55989_gca_000026245
Escherichia_coli_apec_o1_gca_000014845
Escherichia_coli_cft073_gca_000007445
Escherichia_coli_chi7122_gca_000307205
Escherichia_coli_ed1a_gca_000026305
Escherichia_coli_etec_h10407_gca_000210475
Escherichia_coli_gca_000784925
Escherichia_coli_gca_000801185
Escherichia_coli_gca_000931565
Escherichia_coli_gca_001420955
Escherichia_coli_gca_001515725
Escherichia_coli_gca_001520815
Escherichia_coli_gca_001612475
Escherichia_coli_gca_001621665
Escherichia_coli_gca_001677475
Escherichia_coli_gca_001721525
Escherichia_coli_gca_001865295
Escherichia_coli_gca_001900535
Escherichia_coli_gca_001902655
Escherichia_coli_gca_001902735
Escherichia_coli_gca_001936315
Escherichia_coli_gca_002011985
Escherichia_coli_gca_002142695
Escherichia_coli_gca_002156845
Escherichia_coli_gca_002164595
Escherichia_coli_gca_002554485
Escherichia_coli_gca_002803805
Escherichia_coli_gca_002846135
Escherichia_coli_gca_002853805
Escherichia_coli_gca_002854065
Escherichia_coli_gca_002860085
Escherichia_coli_gca_002903105
Escherichia_coli_gca_002925525
Escherichia_coli_gca_003122105
Escherichia_coli_gca_003194125
Escherichia_coli_gca_003203755
Escherichia_coli_gca_003204155
Escherichia_coli_gca_003204955
Escherichia_coli_gca_003254065
Escherichia_coli_gca_003342715
Escherichia_coli_gca_003402955
Escherichia_coli_gca_003413625
Escherichia_coli_gca_003571665
Escherichia_coli_gca_003571785
Escherichia_coli_gca_003627855
Escherichia_coli_gca_003769125
Escherichia_coli_gca_003790525
Escherichia_coli_gca_003812945
Escherichia_coli_gca_003856655
Escherichia_coli_gca_003856675
Escherichia_coli_gca_003966425
Escherichia_coli_gca_003966445
Escherichia_coli_gca_003991155
Escherichia_coli_gca_004011015
Escherichia_coli_gca_004114395
Escherichia_coli_gca_004135815
Escherichia_coli_gca_004135855
Escherichia_coli_gca_004135915
Escherichia_coli_gca_004295365
Escherichia_coli_gca_004358365
Escherichia_coli_gca_004564175
Escherichia_coli_gca_004771235
Escherichia_coli_gca_005221885
Escherichia_coli_gca_005508805
Escherichia_coli_gca_005890115
Escherichia_coli_gca_005954605
Escherichia_coli_gca_005954625
Escherichia_coli_gca_005954725
Escherichia_coli_gca_006088875
Escherichia_coli_gca_006337025
Escherichia_coli_gca_006351885
Escherichia_coli_gca_006352265
Escherichia_coli_gca_006364695
Escherichia_coli_gca_006370475
Escherichia_coli_gca_900184875
Escherichia_coli_gca_900447915
Escherichia_coli_gca_900448155
Escherichia_coli_gca_900448165
Escherichia_coli_gca_900448265
Escherichia_coli_gca_900448335
Escherichia_coli_gca_900448355
Escherichia_coli_gca_900448615
Escherichia_coli_gca_900448835
Escherichia_coli_gca_900448935
Escherichia_coli_gca_900448955
Escherichia_coli_gca_900448985
Escherichia_coli_gca_900449035
Escherichia_coli_gca_900449095
Escherichia_coli_gca_900449115
Escherichia_coli_gca_900449125
Escherichia_coli_gca_900449225
Escherichia_coli_gca_900449295
Escherichia_coli_gca_900449385
Escherichia_coli_gca_900449435
Escherichia_coli_gca_900449455
Escherichia_coli_gca_900449515
Escherichia_coli_gca_900449615
Escherichia_coli_gca_900449865
Escherichia_coli_gca_900449925
Escherichia_coli_gca_900449985
Escherichia_coli_gca_900450185
Escherichia_coli_gca_900450225
Escherichia_coli_gca_900450415
Escherichia_coli_gca_900450445
Escherichia_coli_gca_900450495
Escherichia_coli_gca_900520285
Escherichia_coli_gca_900520345
Escherichia_coli_gca_900520385
Escherichia_coli_gca_900607665
Escherichia_coli_gca_900607725
Escherichia_coli_gca_900636225
Escherichia_coli_gca_900636275
Escherichia_coli_gca_901733115
Escherichia_coli_iai39_gca_000026345
Escherichia_coli_kte100_gca_000408565
Escherichia_coli_nccp15648_gca_003433275
Escherichia_coli_o103_h2_gca_005037795
Escherichia_coli_o103_h2_str_12009_gca_000010745
Escherichia_coli_o104_h4_str_2011c_3493_gca_000299455
Escherichia_coli_o111_h_str_11128_gca_000010765
Escherichia_coli_o111_nm_gca_005037805
Escherichia_coli_o121_h19_gca_005037715
Escherichia_coli_o127_h6_gca_900149915
Escherichia_coli_o127_h6_str_e2348_69_gca_000026545
Escherichia_coli_o139_h28_str_e24377a_gca_000017745
Escherichia_coli_o145_h28_str_rm12581_gca_000671295
Escherichia_coli_o145_nm_gca_005037815
Escherichia_coli_o145_str_rm9872_gca_003586065
Escherichia_coli_o157_gca_002208865
Escherichia_coli_o157_h7_gca_005037735
Escherichia_coli_o157_h7_str_edl933_gca_000006665
Escherichia_coli_o157_h7_str_sakai_gca_000008865
Escherichia_coli_o25b_h4_gca_001874485
Escherichia_coli_o25b_h4_gca_005670575
Escherichia_coli_o25b_h4_gca_005670585
Escherichia_coli_o25b_h4_gca_005670595
Escherichia_coli_o25b_h4_gca_005670875
Escherichia_coli_o25b_h4_gca_005670925
Escherichia_coli_o25b_h4_gca_005671055
Escherichia_coli_o25b_h4_gca_005671075
Escherichia_coli_o25b_h4_gca_005671115
Escherichia_coli_o25b_h4_gca_005671145
Escherichia_coli_o25b_h4_gca_005671155
Escherichia_coli_o25b_h4_gca_005671165
Escherichia_coli_o25b_h4_gca_005671235
Escherichia_coli_o25b_h4_gca_005671255
Escherichia_coli_o25b_h4_gca_005671285
Escherichia_coli_o25b_h4_gca_005673435
Escherichia_coli_o25b_h4_st131_gca_000285655
Escherichia_coli_o26_h11_gca_005037725
Escherichia_coli_o55_h7_str_cb9615_gca_000025165
Escherichia_coli_o7_k1_str_ce10_gca_000227625
Escherichia_coli_o83_h1_str_nrg_857c_gca_000183345
Escherichia_coli_o91_h21_gca_005037775
Escherichia_coli_pcn033_gca_000219515
Escherichia_coli_s88_gca_000026285
Escherichia_coli_se11_gca_000010385
Escherichia_coli_sms_3_5_gca_000019645
Escherichia_coli_str_k_12_substr_mg1655_gca_000005845
Escherichia_coli_str_k_12_substr_w3110_gca_000010245
Escherichia_coli_umn026_gca_000026325
Escherichia_coli_umnf18_gca_000220005
Escherichia_coli_umnk88_gca_000212715
Escherichia_coli_uti89_gca_000013265
Escherichia_coli_w_gca_000184185
Escherichia_coli_xuzhou21_gca_000262125

Supplementary Table 2: The names of each of the 169 *Escherichia coli* genomes used in the pangenome analysis.

Model Organism	# Genes	# URs	Longest UR	Mean / Median UR Length [**SD**]
B. subtilis	4,133	2,711	1,307	161.80/126.00 [**137.73**]
C. crescentus	3,875	2,321	3,377	160.41/121.00 [**172.78**]
E. coli	4,257	2,743	6,175	225.73/144.00 [**353.32**]
M. genitalium	559	157	4,822	287.46/142.00 [**673.23**]
P. fluorescens	5,266	3,509	19,988	261.81/144.00 [**633.71**]
S. aureus	2,556	1,666	2,491	262.87/207.00 [**235.19**]

Supplementary Table 3: The results of running UR-Extractor on the Ensembl annotations for the six model organisms. Lengths presented are in nt and are without the 50 nt extension at each end. Standard deviation is abbreviated as [**SD**].

Model Organism	# Genes	# URs	Longest UR	Mean / Median UR Length [**SD**]
B. subtilis	4,016	2,619	6,159	182.78/125.00 [**311.45**]
C. crescentus	3,704	2,394	6,494	182.41/131.00 [**250.03**]
E. coli	4,263	2,734	3,955	205.00/142.00 [**247.20**]
M. genitalium	995	636	2,546	205.92/133.00 [**221.21**]
P. fluorescens	5,421	3,524	4,164	193.42/139.00 [**239.26**]
S. aureus	2,534	1,650	12,232	274.03/203.00 [**492.37**]

Supplementary Table 4: This table presents the results of running UR-Extractor on the Prodigal CDS predictions for the six model organisms. Lengths presented are in nt and are without the 50nt extension at each end. Standard deviation is abbreviated as [**SD**].

Model Organism	# StORFs	Recovered [**Non-vitiated**]
B. subtilis	2,723	16 [**51**]
C. crescentus	1,997	46 [**100**]
E. coli	3,114	34 [**72**]
M. genitalium	653	2 [**6**]
P. fluorescens	3,465	29 [**51**]
S. aureus	2,354	11 [**16**]

Supplementary Table 5: This table contains the number of Prodigal StORFs and the number of non-vitiated Ensembl genes recovered by StORF-Reporter which Prodigal missed. Non-vitiated genes are those which had an overlap of less than 50 nt with a Prodigal predicted CDS, thus allowing for them to be included in an extracted UR.

Model Organism	Swiss-Prot Hits [**Subject Hit >=80%**]	Intra-Genome Hits [**Subject Hit >=80%**]
B. subtilis	46 [**31**]	38 [**33**]
C. crescentus	7 [**5**]	61 [**47**]
E. coli	75 [**52**]	32 [**28**]
M. genitalium	182 [**5**]	180 [**2**]
P. fluorescens	13 [**5**]	42 [**33**]
S. aureus	19 [**1**]	23 [**13**]

Supplementary Table 6: The table contains the number of Prodigal StORFs which were reported with a hit to either the SwissProt or Intra-Genome protein database. Intra-Genome is the proteome of the same model organism. DIAMOND blastp hits are recorded with a minimum of a 60 bit score and in bold are reported with a subject coverage of 80%.

Data	Unannotated Regions	StORFs
Number of Sequences	563,263	579,661
Median Number Per Genome	3,298	3,345
Longest Sequence (nt)	11,450	9,456
Median Sequence Length (nt) [**Std**]	230 [**210.11**]	135 [**93.13**]

Supplementary Table 7: The numbers and lengths of the unannotated regions (URs) and StORFs extracted from the 169 *Escherichia coli* genomes are presented here. Although there was variability in the genome quality across this set of genomes, the numbers reported here are similar to those reported for the 6 model organisms.

Data	Unannotated Regions	StORFs
Number of Sequences	13,656,918	13,197,690
Median Number Per Genome	2,589	2,432
Longest Sequence (nt)	95,592	47,790
Median Sequence Length (nt) [**Std**]	238 [**301.39**]	141 [**160.10**]

Supplementary Table 8: The numbers and lengths of unannotated regions (UR) and StORFs extracted from the 5,109 genomes of Ensembl Bacteria are presented here. While there was variability in the genome quality across this set of genomes, the numbers reported here are similar to those reported for the 6 model organisms and the *E. coli* pangenome analysis.

	Genome Triplet Abundance			Prodigal Gene Stop Usage				Prodigal StORF Stop Usage			
Genomes	TGA [%]	TAG [%]	TAA [%]	TGA [%]	TAG [%]	TAA [%]	$\tilde{\chi}^2$ p-value	TGA [%]	TAG [%]	TAA [%]	$\tilde{\chi}^2$ p-value
B. subtilis	180,347 [**49.57**]	52,378 [**14.39**]	131,084 [**36.03**]	932 [**23.21**]	563 [**14.02**]	2,521 [**62.77**]	<0.00001	1,123 [**41.24**]	465 [**17.08**]	1,135 [**41.68**]	<0.00001
C. crescentus	102,367 [**69.85**]	32,635 [**22.27**]	11,541 [**7.87**]	1,735 [**46.84**]	1,230 [**33.21**]	739 [**19.95**]	<0.00001	1,176 [**58.88**]	488 [**24.44**]	333 [**16.68**]	<0.00001
E. coli	164,560 [**46.64**]	53,119 [**15.05**]	135,187 [**38.31**]	1,232 [**28.90**]	332 [**7.79**]	2,699 [**63.31**]	<0.00001	1,149 [**36.90**]	521 [**16.73**]	1,444 [**46.37**]	<0.00001
M. genitalium	25,382 [**28.26**]	18,982 [**21.13**]	45,456 [**50.60**]	612 [**61.51**]	110 [**11.06**]	273 [**27.44**]	<0.00001	263 [**40.28**]	127 [**19.45**]	263 [**40.28**]	<0.00001
P. fluorescens	189,251 [**64.36**]	56,411 [**19.18**]	48,377 [**16.45**]	3,010 [**55.52**]	770 [**14.20**]	1,641 [**30.27**]	<0.00001	1,712 [**49.41**]	806 [**23.26**]	947 [**27.33**]	<0.00001
S. aureus	119,798 [**30.87**]	73,821 [**19.02**]	194,474 [**50.11**]	268 [**10.58**]	379 [**14.96**]	1,886 [**74.46**]	<0.00001	562 [**23.87**]	455 [**19.32**]	1,337 [**56.80**]	<0.00001

Supplementary Table 9: Presented in this table are the following: the triplet abundance of the three canonical stop codons found throughout the six model organism genomes (totaled from both forward and reverse strands), the stop codons used in the Prodigal predicted CDS genes, and the end stop codon used in the StORFs identified from within the URs reported by Prodigal, both from the 6 model organisms which have been inspected. A chi squared test was performed on each model organism: triplet abundance vs Prodigal gene stop codon usage and triplet abundance vs StORF stop codon. Each test resulted in a rounded p-value of <0.00001.

Genomes	Genome Triplet Abundance			Ensembl Gene Stop Usage				Ensembl StORF Stop Usage			
	TGA [%]	TAG [%]	TAA [%]	TGA [%]	TAG [%]	TAA [%]	$\tilde{\chi}^2$ p-value	TGA [%]	TAG [%]	TAA [%]	$\tilde{\chi}^2$ p-value
B. subtilis	180,347 **[49.57]**	52,378 **[14.39]**	131,084 **[36.03]**	927 **[23.11]**	560 **[13.96]**	2,524 **[62.93]**	<0.00001	1,063 **[41.17]**	415 **[16.07]**	1,104 **[42.76]**	<0.00001
C. crescentus	102,367 **[69.85]**	32,635 **[22.27]**	11,541 **[7.87]**	1,770 **[47.36]**	1,225 **[32.78]**	742 **[19.86]**	<0.00001	1,021 **[59.81]**	412 **[24.14]**	274 **[16.01]**	<0.00001
E. coli	164,560 **[46.64]**	53,119 **[15.05]**	135,187 **[38.31]**	1,151 **[28.41]**	279 **[6.89]**	2,621 **[64.70]**	<0.00001	1,153 **[37.98]**	474 **[15.61]**	1,409 **[46.41]**	<0.00001
M. genitalium	25.382 **[28.26]**	18.982 **[21.13]**	45.456 **[50.60]**	0 **[0]**	129 **[27.10]**	347 **[72.90]**	<0.00001	76 **[42.22]**	26 **[14.44]**	78 **[43.33]**	<0.00001
P. fluorescens	189,251 **[64.36]**	56,411 **[19.18]**	48,377 **[16.45]**	2,869 **[55.41]**	734 **[14.18]**	1,575 **[30.42]**	<0.00001	1,860 **[51.20]**	815 **[22.43]**	958 **[26.37]**	<0.00001
S. aureus	119,798 **[30.87]**	73,821 **[19.02]**	194,474 **[50.11]**	271 **[10.84]**	379 **[15.16]**	1,850 **[74.00]**	<0.00001	540 **[23.60]**	411 **[17.96]**	1,338 **[58.45]**	<0.00001

Supplementary Table 10: Presented in this table are the following: the triplet abundance of the three canonical stop codons found throughout the six model organism genomes (totalled from both forward and reverse strands), the stop codons used in the Ensembl annotated CDS genes, and both end stop codons used in the StORFs identified from within the URs reported by Ensembl, both from the 6 model organisms which have been inspected. A chi-squared test was performed on each model organism: triplet abundance vs Ensembl gene stop codon usage and triplet abundance vs StORF stop codon. Each test resulted in a rounded p-value of <0.00001.

3 Processing

Listed below are the parameters used in the extraction of URs, StORFs and CD-Hit sequence clustering. The full set of parameters for UR-Extractor, StORF-Finder and StORF-Reporter, including the default options which were not modified are available on the StORF-Reporter Github repository (https://github.com/NickJD/StORF-Reporter)

3.1 UR-Extractor User Menu

```
UR-Extractor -f Ensembl_Genome.fasta -gff Ensembl_Genome.gff3 -oname
    Ensembl_Genome_UR -gz True
```

Listing 1: Example parameters used for extracting URs from Ensembl annotations with UR-Extractor.

3.2 StORF-Finder User Menu

```
StORF-Finder -f Ensembl_Genome_UR.fasta -aa True -gff -oname
    Ensembl_Genome_UR_StORFs
```

Listing 2: Example parameters used for extracting StORFs from the URs of Ensembl genomes.

3.3 StORF-Reporter User Menu

```
StORF-Reporter -anno Ensembl Single_Genome -p Ensembl_Genome.fasta -oname
    Ensembl_Genome_UR -gz True
```

Listing 3: Example parameters for StORF-Reporter to produce an enhanced annotation from Ensembl genomes.

3.4 CD-Hit Clustering

```
cd-hit -i Escherichia_coli_PEP.fa -o Escherichia_coli_PEP.fa_CD_c90_s60 -c 0.9
-s 0.6 -sc 1 -sf 1 -p 1 -g 1 -d 0 -M 10000 -T 8
```

Listing 4: Listed here are the parameters used for the CD-HIT sequence clustering. Each clustering round was performed with the same parameters for both the Ensembl representatives and the inclusion of the StORF sequences, for both the cross-genera and *E. coli* pangenome analysis.
